# Stapled peptide inhibitors of RAB25 target context-specific phenotypes in cancer

**DOI:** 10.1038/s41467-017-00888-8

**Published:** 2017-09-22

**Authors:** Shreya Mitra, Jeffrey E. Montgomery, Matthew J. Kolar, Gang Li, Kang J. Jeong, Bo Peng, Gregory L. Verdine, Gordon B. Mills, Raymond E. Moellering

**Affiliations:** 1 Department of Systems Biology, University of Texas M.D. Anderson Cancer Center, Houston, TX 77030 USA; 2 Department of Chemistry, The University of Chicago, Chicago, IL 60637 USA; 3 Institute for Genomics and Systems Biology, The University of Chicago, Chicago, IL 60637 USA; 4 Department of Chemistry and Chemical Biology, Harvard University, Cambridge, MA 02138 USA; 5 Department of Stem Cell and Regenerative Biology, Harvard University, Cambridge, MA 02138 USA; 6 Department of Bioinformatics and Computational Biology, University of Texas M.D. Anderson Cancer Center, Houston, TX 77030 USA

## Abstract

Recent evidence has established a role for the small GTPase RAB25, as well as related effector proteins, in enacting both pro-oncogenic and anti-oncogenic phenotypes in specific cellular contexts. Here we report the development of all-hydrocarbon stabilized peptides derived from the RAB-binding FIP-family of proteins to target RAB25. Relative to unmodified peptides, optimized stapled peptides exhibit increased structural stability, binding affinity, cell permeability, and inhibition of RAB25:FIP complex formation. Treatment of cancer cell lines in which RAB25 is pro-oncogenic with an optimized stapled peptide, RFP14, inhibits migration, and proliferation in a RAB25-dependent manner. In contrast, RFP14 treatment augments these phenotypes in breast cancer cells in which RAB25 is tumor suppressive. Transcriptional profiling identified significantly altered transcripts in response to *RAB25* expression, and treatment with RFP14 opposes this expression profile. These data validate the first cell-active chemical probes targeting RAB-family proteins and support the role of RAB25 in regulating context-specific oncogenic phenotypes.

## Abstract (web summary)

The Ras-family small GTPase RAB25 can exert both pro- and anti-oncogenic functions. Here, the authors develop all-hydrocarbon stabilized peptides targeting RAB25 and influencing the context-specificity phenotypes in cancer cell lines.

## Introduction

RAB proteins are members of the Ras-oncogene superfamily of small GTPases and are broadly involved in membrane trafficking events^1, 2^. Members of the RAB11 subfamily, which include RAB11a/b and RAB25, have been shown to play roles in compartmentalization of early endosomes^2^ as well as trafficking, localization, and recycling of integral membrane proteins and receptors in polarized cells. Several studies have implicated RAB proteins^3, 4^, and specifically RAB25, in promoting the pathogenesis of cancers of the liver^5^, breast^6^, and ovary^6^. More generally, deregulation of endocytosis, vesicular transport and receptor trafficking appears to be an emerging hallmark in cancer^3^. Constitutive RAB25 activity is attributed to a glutamine-to-leucine substitution at position 70 in its GTP-binding domain, compared to other RAB-family members, and has been mechanistically linked to oncogenic phenotypes through activation of AKT signaling^6^, protection against metabolic stress^7^, and recycling of receptor tyrosine kinases^8, 9^ and α5β1 integrins^8, 10, 11^. The molecular underpinnings of these phenotypes are poorly understood at present and, paradoxically, recent literature has also implicated RAB25 as a tumor suppressor that is silenced in invasive breast cancers^12, 13^, colon cancer^14^ and intestinal neoplasias^15^. In light of the causative associations observed between RAB25 signaling and malignant phenotypes in cell lines, animal models and humans, development of RAB25 inhibitors is desirable for their potential utility as therapeutics. The creation of first-in-class chemical probes targeting these proteins would also enable mechanistic evaluation of the diverse roles of RAB25 in cancer as well as aid in unraveling the many signaling pathways involving RAB proteins in diverse biological contexts.

**Fig. 1:**
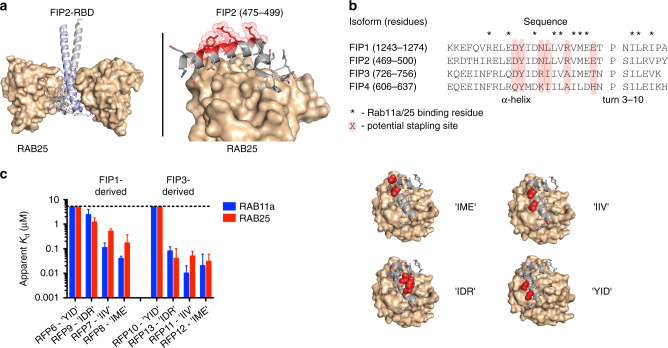
Development of stapled peptide ligands targeting RAB11a and RAB25. **a** Crystal structure of the RAB25:FIP2 heterotetramer (*left*). The C-terminal RAB-binding domain of FIP2 binds as a helix-turn-helix peptide (*gray* and *blue*) to a hydrophobic groove on RAB25 (*tan surface*). Side chains not involved in RAB25 binding are highlighted as *red surfaces* while others are shown as sticks (*right*). PDB accession: 3TSO. **b** Sequence alignment of the RAB-binding domains from Class-I (FIP1 and 2) and Class-II (FIP3 and 4) FIP proteins. Residue annotations were assigned based on published RAB11a:FIP3 and RAB25:FIP2 crystal structures. **c** Graphical depiction of apparent affinities for initial series of FIP1- and FIP3-derived stapled peptides for binding to both RAB11a and RAB25. Each peptide stapling position is denoted by the residues in FIP3 spanned by the hydrocarbon staple (e.g., “IIV”), highlighted in *red* on the schematic structures shown (*right*). The affinity *graphs* show the mean apparent *K*_d_ with *error bars* representing the 95% confidence interval from triplicate replicates and application of a sigmoidal curve fit using Prism 5 software

Members of the RAB11-family of interacting proteins (Rab11-FIPs, referred to herein as FIPs), which are subdivided into Class-I (FIP1, FIP2, and FIP5) and Class-II (FIP3, 4) proteins, have been shown to be obligate members of RAB11/25 trafficking complexes^16, 17^. Biochemical studies, which have primarily focused on RAB11 isoforms, have established that FIP proteins engage RAB11 and RAB25 through a conserved C-terminal RAB-binding domain (RBD)^18, 19^, which in several X-ray structures exists in an extended α-helix-turn-3_10_-helix conformation that contacts a hydrophobic groove on RAB25 (Fig. 1a). These studies also indicate that RAB/FIP complexes exist, at least in vitro, as heterotetramers, with extensive RAB-FIP and FIP-FIP contacts mediating complex stability (Fig. 1a, b). Overexpression of dominant-negative mutant FIP proteins that are incapable of binding RAB11/25, as well as shRNA knockdown of *FIP* expression have been shown to functionally block recruitment of cargo proteins to RAB11 and/or RAB25 in cells^8, 9^. In light of these data, we reasoned that development of molecules targeting the RAB25:FIP binding interface could enable pharmacologic disruption of RAB25 and/or RAB11 signaling in cells. Here we report the design and synthesis of all-hydrocarbon stapled peptides that exhibit increased structural stability and binding affinity toward RAB25. Several optimized cell permeable stapled peptides disrupt RAB25:FIP complex formation in vitro and in situ, and oppose the context-specific phenotypes associated with RAB25 function in ovarian and breast cancer cell lines.

## Results

### Design, synthesis and RAB11/25 binding of RFP stapled peptides

Due the general difficulty in targeting protein−protein interactions with small molecules, as well as the α-helical interaction motif of the FIP-RBD, we hypothesized that the RAB-FIP interface might be a suitable system for targeting by all-hydrocarbon stapled α-helical peptides^20^, which have proven successful in targeting diverse intracellular protein−protein interactions^21–28^. Sequence alignment of the C-terminal RBDs from FIP1-4 highlighted conserved residues that contact the RAB11a and RAB25 surface in X-ray structures with FIP3 and FIP2, respectively, as well as orthogonal positions in the RBD α-helix that might be suitable for incorporation of stapling residues (Fig. 1b). A representative panel of stapled peptides (Restrained-FIP Peptides, RFPs) containing a single *i *→* i + *4 hydrocarbon staple at each of four suitable positions were designed from Class-I (FIP1) and Class-II (FIP3) sequences (Fig. 1c). Stapled peptides, as well as unmodified peptides encompassing the RBDs of FIP proteins, were synthesized with an N-terminal monoethylene glycol moiety linked to a fluoresceinisothiocyanate (FITC) label, which enabled fluorescence polarization binding affinity measurements with recombinant RAB11a (activated Q70L mutant, herein referred to as RAB11a) and RAB25 proteins. Unmodified Class-I peptides (FIP1 and 2) showed essentially no binding to either RAB11a or RAB25 over the concentrations tested, while those from Class-II (FIP3 and 4) displayed micromolar affinities (Supplementary Table 1). Despite the low relative affinities observed, the RAB11a-FIP interaction appeared to be quite specific as a molecular change as subtle as oxidation of a methionine involved in binding (RFP5, a separable byproduct of chemical synthesis) resulted in ~ fivefold lower binding affinity for RAB11a. In contrast to the unmodified peptides tested, a wide range of affinities was observed for RAB11a and RAB25 within the panel of stapled peptides. In line with the results from unmodified peptides, Class-II-derived stapled peptides (RFP10−13) generally displayed stronger binding toward both RAB11a and RAB25 than those from Class-I sequences (RFP6−9). Several structure-activity relationships with regard to staple position were observed in both classes (Supplementary Table 1 and Fig. 1c). Both peptides containing the most N-terminal staple position (RFP6 and 10, spanning residues ‘YID’ in FIP3, thus referred to as the ‘YID’ staple position) displayed negligible affinity toward RAB11a and RAB25, which is likely caused by a steric clash between the hydrocarbon bridge and Ala75 and Ala76 in RAB11a and RAB25, respectively (Supplementary Fig. 1a, b). Stapled peptides containing the internal staple positions ‘IME’, ‘IIV’, and ‘IDR’ displayed apparent *K*_d_ values that were significantly lower (up to ~ 100-fold) than their unmodified counterparts—highlighting the positive effect of hydrocarbon stapling on target engagement (Fig. 1c). Finally, peptides from both FIP classes generally displayed higher binding affinity for RAB11a than RAB25, which, to our knowledge, has not been systematically explored or reported in the literature.

### Stability and binding affinity are improved and correlated in optimized RFPs

**Fig. 2:**
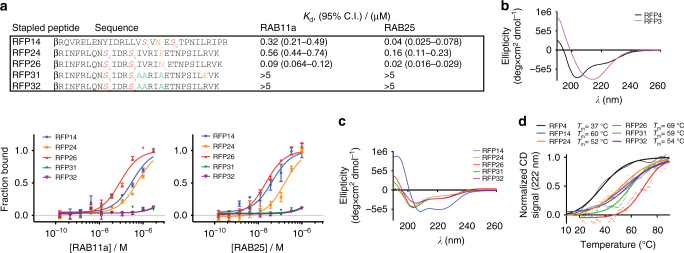
Optimized stapled peptides targeting RAB25 show correlated structural stability and high-affinity binding. **a** Fluorescence polarization binding curves for three lead compounds, RFP14, 24, and 26, as well as two negative control peptides, RFP31 and 32, to both RAB11a and RAB25 (*bottom*). Sequences and calculated apparent *K*_d_ values are shown (*top*). Residues highlighted in *yellow* denote potential gain-of-function mutations to RAB-binding positions, while those in *green* denotes loss-of-function alanine replacement of hydrophobic RAB-binding side chains for the negative controls. **b** Circular dichroism (CD) spectra of unmodified peptides derived from the RBD of FIP3 and FIP4. **c** CD spectra of optimized RFP stapled peptides. **d** Thermal denaturation CD curves measuring relative helical content (CD absorbance at 222 nm) of the indicated peptides over a temperature range from 10 to 90 °C. Individual data points at one-degree increments are shown with a sigmoidal curve fit overlay. Binding *data points* represent the mean ± s.e.m. from triplicate measurements. Affinities listed represent the mean apparent *K*_d_ with 95% confidence interval from triplicate replicates and application of a sigmoidal curve fit using Prism 5 software. β, beta-alanine

Several stapled peptides in the original panel showed significantly improved binding affinity for both RAB11a and RAB25 relative to their unmodified counterparts, and we therefore sought to further optimize these leads for several properties, including: chemical stability, cell permeability, solubility, and isoform-selectivity. Chemical stability was accomplished by replacing the oxidation-sensitive critical methionine present in wild-type FIP1-3 (e.g., M489 in the RAB25:FIP2 structure; Supplementary Fig. 1c) with a norleucine (N_L_) isostere or other hydrophobic residue. Improved solubility and cell permeability were aided by replacing non-binding, acidic residues with neutral-polar or cationic residues, as well as incorporation of additional charged residues on the periphery of the stapled peptide to generate a net-positive charge^29^. Candidate stapled peptides were iteratively synthesized and screened for binding against RAB11a and RAB25 by fluorescence polarization. Three optimized peptides from the ‘IME’ and ‘IDR’ staple positions, RFP14, RFP24, and RFP26, emerged from this optimization process and exhibited tight binding with a preference for RAB25 relative to RAB11a (Fig. 2a). As controls for biochemical and cell-based assays, we also synthesized two inactive stapled peptides, RFP31 and RFP32, which contained alanine-substitution of three critical hydrophobic FIP residues involved in RAB11a/25 binding (L485, V486, and M489 in FIP2; Supplementary Fig. 1c). Stapled peptide formal charge has been shown to correlate with active cell uptake in several studies^22–24, 30–32^, and therefore the two negative controls RFP31 and RFP32 differed by a single Glu-to-Arg mutation to span the range of formal charges in the active lead compounds (Fig. 2a). Relative to the active leads, RFP31 and RFP32 showed marked reductions in binding affinity (*K*_d_ > 5 μM) for both RAB11a and RAB25, further supporting the specificity of the binding interaction between RFP peptides and RAB11a/25.

To probe the effect of hydrocarbon stapling on the stabilization of peptide secondary structure, we employed circular dichroism spectroscopy (CD). Unmodified FIP3 and FIP4 peptides (RFP3 and RFP4) generated spectra characteristic of β-sheet and partially disordered peptide folds, respectively (Fig. 2b). RFP24, 26, 31, and 32 generated spectra that were more α-helical than their unmodified FIP3 parent sequence as measured by CD signal at 222 nm (Fig. 2c). RFP14 exhibited a classic α-helical CD spectrum with strong minima at ~ 208 and 222 nm (Fig. 2c). To further test the stabilizing effect of hydrocarbon stapling, especially considering that the bioactive conformation of FIP peptides is not expected to be entirely α-helical, we measured the thermal stability of several unmodified and stapled peptides by CD. Although the unmodified FIP4 peptide showed modest helical character by CD (Fig. 2b), it exhibited low thermal stability as evidenced by a *T*_m_-value of 37 °C (Fig. 2d). All optimized hydrocarbon stabilized RFPs showed significantly higher thermal stability, with *T*_m_-values ranging from 52 to 69 °C (Fig. 2d). Together, these data confirm that peptide stapling results in stabilization of FIP peptide secondary structure, which is correlated with significant gains in affinity toward both RAB11a and RAB25.

### Cell permeable RFP peptides inhibit RAB25:FIP complex formation

**Fig. 3:**
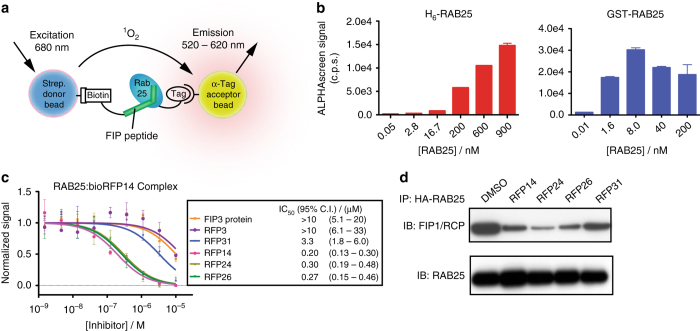
RFPs form stable complexes with RAB25 and compete for FIP binding. **a** Schematic of the RAB25 ALPHAscreen proximity assay. Incubation of a synthetic, biotinylated-FIP-derived peptide with tagged (His_6_- or GST-) RAB25 leads to the formation of the soluble complex and proximal association of streptavidin-coated donor beads with acceptor beads (Ni^2+^-NTA or anti-GST). Donor bead excitation at 680 nm produces singlet oxygen, which selectively initiates a luminescent cascade in bound acceptor beads. **b** Bio-RFP14 binds H_6_- (*left*) and GST-RAB25 (*right*) in a dose-dependent manner, leading to robust ALPHAscreen signal. **c** Competitive inhibition of RAB25:RFP14 complex formation by soluble RFP and FIP peptide ligands. Constant H_6_-RAB25 (0.2 μM) and bio-RFP14 (0.2 μM) were incubated in the presence of indicated ligand concentrations prior to addition of ALPHAscreen reagents and reading of luminescent signal. **d** RFP stapled peptides (1 μM) compete with HA-RAB25 binding and co-immunoprecipitation of FIP1 in HEY RAB25 cell lysate. These results are representative of experiments performed in triplicate. Binding *data points* represent the mean ± s.e.m. from triplicate measurements. IC_50_ values represent mean with 95% confidence interval from triplicate replicates and application of a sigmoidal curve fit using Prism 5 software

We next sought to directly measure the formation of RAB25:RFP complexes as well as functional antagonism of the RAB25:FIP binding interaction by RFP stapled peptides. We developed an ALPHAscreen proximity assay to measure RAB25:FIP complexes in solution^33^. Unmodified or stapled RFP peptides with an N-terminal biotin moiety were used in combination with recombinant RAB25 proteins harboring affinity tags (e.g., His_6_- or GST-), which, when associated in solution, localizes acceptor and donor beads in proximity to initiate a sensitive and specific luminescent signal (Fig. 3a). Biotin-labeled RFP14 (bio-RFP14) strongly associated with both His_6_-labeled and GST-labeled RAB25 (Fig. 3b), which varied in saturation point due to the different binding capacities of anti-GST and anti-H_6_ beads. Using this assay in a competitive format, we next measured the relative potency of soluble RFPs and unmodified peptides to inhibit complex formation. While FIP3 peptide (residues 726–756) and protein (residues 649–756) exhibited some inhibition of complex formation at high concentrations (Fig. 3c), the stapled peptides RFP14, 24, and 26 all showed potent inhibition of complex formation (Fig. 3c). The mutant RFP31 compound exhibited significantly reduced inhibitory potential. Finally, to test whether RFPs inhibit complex formation with full-length endogenous RAB25 and FIP proteins, we performed competitive pull-down assays in lysate from HEY cells expressing HA-RAB25 and H_6_-FIP1. Immunoprecipitation of HA-RAB25 effectively co-precipitated FIP1, and this interaction was significantly blunted by treatment with RFP14, RFP24, and RFP26 (Fig. 3d), whereas RFP31 was less effective. Together, these data confirm that RFP stapled peptides inhibit RAB25:FIP complex formation.

As a final filter for RFP optimization, confocal fluorescence microscopy was used to evaluate RFP cellular penetration in MCF7 breast cancer cells, which express RAB25. Incubation with 5 μM fluorescein-labeled RFPs for 8 h revealed that RFP14, 24, 26, 31, and 32 each showed cellular uptake and distribution in the cytosol (Supplementary Fig. 2). Despite bearing a formal charge between the other optimized RFP stapled peptides ( + 2) and sharing the ‘IDR’ structural backbone, RFP31 showed modest cellular uptake and punctate distribution relative to other compounds (Supplementary Fig. 2). With a formal charge of  + 1, RFP14 exhibited significant cellular uptake and distribution throughout the cytosol and nucleus of MCF7 cells. RFP24, RFP26, and RFP32, all derived from the ‘IDR’ structural backbone, showed high levels of intracellular fluorescence (Supplementary Fig. 2). Importantly, RFP31 and RFP32 showed equivalently reduced binding to both RAB11a and RAB25 in vitro (Fig. 2a), however due to the superior cellular uptake of RFP32, it was chosen to be the comparator for cellular studies. Together with the active compounds RFP14, RFP24, and RFP26, which encompass a range of target binding affinities with roughly equivalent, robust cell penetration, this panel of stapled peptides were ideal candidates to study RAB25-mediated phenotypes in cells.

### RFP14 inhibits RAB25-dependent phenotypes in cancer cells

**Fig. 4:**
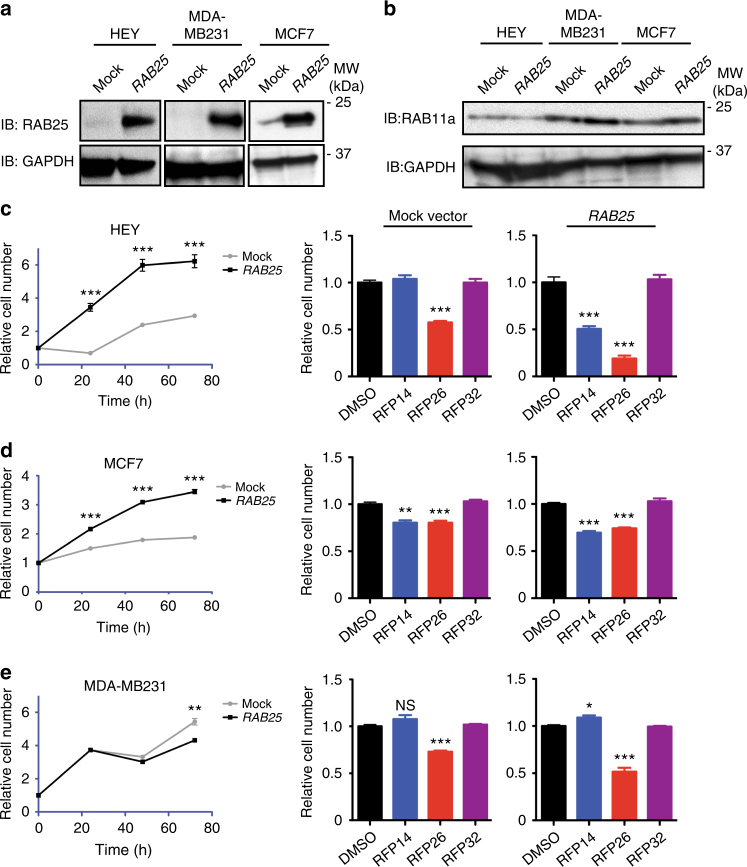
RFP peptides alter RAB25-driven cell proliferation. **a** Western blot analysis of mock vector and *RAB25* overexpressing HEY, MDA-MB231, and MCF7 cells. **b** Western blot analysis for endogenous RAB11a in HEY, MDA231 and MCF7 isogenic lines with or without RAB25 overexpression. **c** *Left panel* compares the viability of untreated HEY cells under low-serum condition in mock or RAB25 expressing lines while *right panel* shows the effects of treatment with DMSO or RFP14, 26, and 32 (15 μM) on the viability of HEY mock or RAB25 expressing cell lines at 48 h post-treatment. **d**, **e** shows corresponding results from MCF7 isogenics and MDA-MB231 isogenics, respectively. RAB25 expression results in enhancement (HEY, MCF7) or inhibition (MDA-MB231) of cell proliferation, which is reversed by RFP14, but not RFP32. Images and data shown represent mean ± s.e.m. of triplicate biological replicates. *P < 0.05; **P < 0.01; ***P < 0.001, Student’s *t*-test. NS, not significant

With the in vitro activity and cell penetration of optimized RFP stapled peptides confirmed, we next sought to determine whether RFP-mediated disruption of RAB25:FIP complexes would antagonize RAB25-mediated phenotypes in cancer cells. Several reports in the literature have established that *RAB25* expression confers either pro-tumorigenic or anti-tumorigenic phenotypes in specific cellular contexts. For example, overexpression of *RAB25* in HEY ovarian cancer and MCF7 breast cancer cell lines has been shown to promote cell proliferation, migration, and other malignant phenotypes^6, 13^. Conversely, overexpression of *RAB25* in aggressive MDA-MB231 triple-negative breast cancer cells, which exhibit hypermethylation of the *RAB25* locus^34^, blunts oncogenic phenotypes, including cell migration and proliferation^13, 34^. In light of these disparate roles, we explored the activity of RFP peptides on RAB25-specific cellular phenotypes under contexts where opposite effects would be expected, increasing the likelihood that any observed changes would be due to RAB25-dependent activity. We generated HEY, MCF7 and MDA-MB231 cell lines stably overexpressing *RAB25* (referred to as RAB25-HEY, RAB25-MCF7, and RAB25-MDA-MB231s). Both HEY and MDA-MB231 cells express very low levels of endogenous RAB25 protein, whereas MCF7 harbors RAB25 at higher endogenous levels (Fig. 4a). In agreement with previous observations, RAB11a is constitutively expressed in all of these lines (Fig. 4b).

We first determined the effect of RAB25 on cell proliferation, and found that *RAB25* overexpression promotes cell growth in HEY cells, in agreement with previous reports (Fig. 4c). For cell-based studies with RFP stapled peptides, we focused on the two most potent active compounds RFP14 and RFP26, as well as the inactive control RFP32. Treatment of control HEY cells with RFP14 and the negative control RFP32 showed no effect on proliferation at 48 h. In contrast, RAB25-HEY cell proliferation was significantly inhibited by RFP14, but not the negative control RFP32 (Fig. 4c). RFP26 inhibited proliferation of both mock and RAB25-HEY cells. MCF7 cells, which endogenously express RAB25, exhibited similar proliferation in mock and RAB25 overexpression conditions (Fig. 4d). In this context, RFP14 and RFP26 inhibited growth in both control and RAB25-MCF7 cells, in agreement with studies showing decreased proliferation of MCF7 cells in which endogenous RAB25 was inhibited by shRNA knockdown^6, 13^. Finally, cell proliferation of MDA-MB231 cells was significantly decreased with RAB25 overexpression relative to mock cells, in agreement with previous assertions of a tumor suppressive role. Treatment of both cell lines with RFP14 led to slightly increased proliferation, an effect that was more pronounced in RAB25 overexpressing cells (Fig. 4e). As was the case with HEY cells, RFP26 decreased MDA-MB231 viability in both contexts, clearly deviating from the RAB25-dependent effects observed with RFP14. These studies established that modulation of RAB25 levels, but not RAB11a, which was constitutively expressed in all cell lines tested, resulted in context-specific and opposing effects on proliferation, all of which were significantly inhibited by RFP14.

**Fig. 5:**
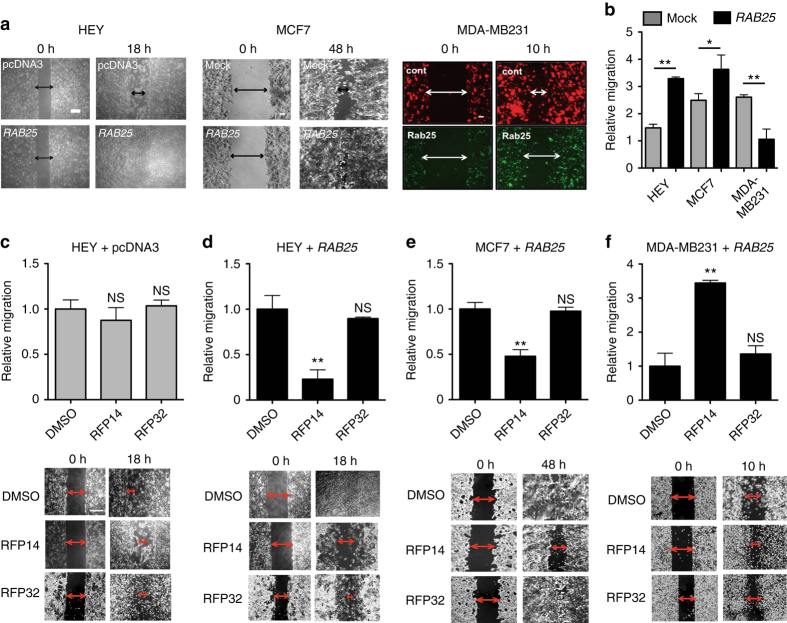
RFP14 specifically inhibits RAB25-dependent migration in cancer cells. **a**, **b** Representative time-dependent scratch-wound cell migration assay images **a** and quantification **b** of mock vector and *RAB25*-expressing HEY, MCF7, and MDA-MB231 cells. RAB25 expression results in significant enhancement (HEY, MCF7) or inhibition (MDA-MB231) of cell migration. These data are representative of triplicate biological replicates. **c**, **d** RFP 14 (10 μM) significantly impaired cell migration of *RAB25*-expressing **d**, but not vector control **c**, HEY cells. **e** RFP14 (10 μM), but not RFP32, significantly impaired cell migration of *RAB25*-expressing MCF7 cells. **f** Treatment of *RAB25*-expressing MDA-MB231 cells with RFP14, conversely, significantly increased cell migration relative to vehicle while the inactive control RFP32 remained unchanged compared to vehicle control. Representative scratch wound images are shown for RFP14, RFP32, and vehicle (DMSO) treatment conditions with overlaid arrows highlighting distance between cell fronts. Images and data shown represent mean ± s.e.m. of triplicate biological replicates. *P < 0.05; **P < 0.01, Student’s *t*-test. NS, not significant. Inset *scale bars* for HEY cells in **a** = 250 μm; MCF7 and MDA-MB231 cells in **a** = 100 μm. Inset *scale bars* for **c**, **d**, **e**, **f** = 250 μm

The most validated RAB25-dependent phenotype in cancer is cell migration. In this context, we again validated opposing RAB25-dependent effects, with significant induction of migration in both HEY and MCF7 cells, and inhibition of migration in MDA-MB231 cells (Fig. 5a, b). Treatment of mock vector expressing HEY cells with RFP peptides (10 μM) did not significantly affect cell migration (Fig. 5c), likely due to the low endogenous levels of RAB25. Treatment of *RAB25* overexpressing HEY and MCF7 cells with RFP14, on the other hand, resulted in significant inhibition of cell migration (Fig. 5d, e). This inhibitory effect was not observed with the inactive control RFP32. Notably, treatment of *RAB25* expressing MDA-MB231 cells with RFP14 resulted in significantly increased cell migration and wound closure, while RFP32 treatment did not have a significant effect (Fig. 5f). In agreement with the proliferation studies above, treatment with RFP26 showed considerable toxicity in all cell lines tested, preventing proper wound healing studies. In combination with the previous data, the unique inhibition of both proliferation and migration by RFP26 in both mock and *RAB25* overexpressing cells may result from a lack of selectivity between RAB11a and RAB25, a notion supported by its high affinity for both isoforms in vitro relative to RFP14 or RFP24 (Fig. 2a). It is important to point out that these RAB25-dependent effects were observed in RAB11a-expressing contexts (Fig. 4b), and thus phenotypic responses stemming from RAB11a inhibition should be apparent and independent of *RAB25* expression. As an additional measure to support target engagement of RAB25 and/or RAB11a in cells, we applied a cellular thermal shift assay (CETSA)^35^ approach to monitor target engagement for RAB25 and RAB11a. Monitoring endogenous RAB25 and RAB11a in MCF7 proteomes revealed no appreciable *T*_m_ shifts with RFP32 treatment, indicating no apparent binding of either isoform (Supplementary Fig. 3). Treatment with RFP14, on the other hand, resulted in a significant + 2.5 degree shift in *T*_m_ for RAB25, but not for RAB11a, indicative of target engagement of a significant fraction of RAB25 in cells but not for RAB11a. These data indicate that the specific effects of RFP14 on RAB25-dependent signaling likely stem from a combination of higher RAB11a abundance, differential regulation of RAB11a signaling complexes in cells, as well as the tighter binding affinity observed between RFP14 and RAB25 relative to RAB11a. Taken together, these biochemical annotations of RFP14, and its ability to confer both loss- and gain-of-function phenotypes in distinct contexts, strongly supports its utility as a RAB25-targeting chemical probe.

**Fig. 6:**
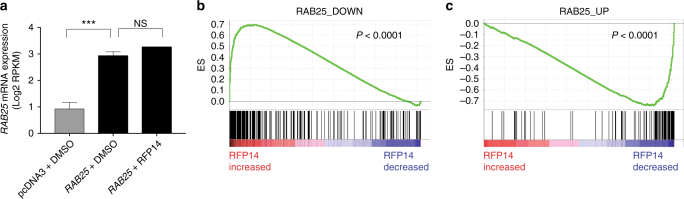
RFP14 inhibits RAB25-dependent gene expression in ovarian cancer cells. **a** Relative *RAB25* mRNA levels in pcDNA3-HEY cells treated with DMSO, *RAB25-*expressing HEY cells treated with DMSO, and *RAB25-*expressing HEY cells treated with RFP14 measured by RNA-Seq profiling. **b**, **c** Quantitative GSEA plots of RAB25_UP and RAB25_DOWN gene set enrichment within the RFP14 vs. DMSO gene expression profile in *RAB25*-expressing HEY cells. Both RAB25-depenent gene sets were significantly enriched in the RFP14 vs. DMSO profile as indicated by the GSEA results for RAB25_DOWN (ES = 0.70, NES = 1.35, *P* < 0.0001) and RAB25_UP (ES = −0.74, NES = −1.61, *P* < 0.0001). The data represents two technical replicates from two independent biological replicate experiments. ES, enrichment score; NES, normalized enrichment score; NS, not significant. ***P < 0.005, Student’s *t*-test

Finally, to compliment the RAB25-dependent phenotypic assays employed here, we also sought to capture the effects of RFP14 treatment on global signaling in a RAB25-dependent context. Specifically, we wanted to identify the gene expression changes enacted by RAB25-dependent signaling, and determine whether these program(s) are affected by RFP14 treatment. To ask this question in a global and unbiased manner, we performed RNAseq on transcripts from pcDNA3-expressing and RAB25-expressing HEY cells. Expression profiling identified a set of 104 and 269 genes that were significantly increased (RAB25_UP gene set) and decreased (RAB25_DOWN gene set) with RAB25 expression, respectively (Supplementary Table 2). In parallel, we performed global profiling of transcripts that changed in response to RFP14 treatment, relative to DMSO, in RAB25-expressing HEY cells. Each RAB25-dependent gene set was queried for enrichment within the expression profile generated by comparing DMSO and RFP14 treated RAB25-HEY cells using GSEA^36^, thereby asking whether RAB25-regulated genes are targeted by RFP14 in an unbiased manner and in the specific biologic contexts associated with phenotypic changes. Both RAB25-dependent gene sets were significantly enriched in the RFP14 gene expression profile but in the opposite direction (i.e., genes upregulated by RAB25 were downregulated by RFP14, Fig. 6 a–c), and leading edge gene ontology analysis identified genes involved in ER and Golgi-mediated vesicular trafficking, small GTPase signaling, transmembrane signaling, GPCR signaling, and cAMP signaling, among other pathways (Supplementary Fig. 5a−d). Targeted expression analysis with qPCR validated significant effects of RFP14, contrasted by minimal effects of the less active control RFP32, on the expression of enriched genes from functional categories associated with protein modification and vesicular trafficking (*MGAT1, PRSS23, LY6K*), migration (*AMIGO3*, *PRSS23*), GPCR signaling (*FPR1*, *GPER1*, *LPAR4, P2RY14*), regulation of transcription (*CEBPG*, *FOXI1*), as well as tumorigenesis (*IGF1*, *LPAR4*, *VHL*; Supplementary Fig. 6; Supplementary Table 4). These expression profiles should provide hypotheses to further explore the downstream pathways enacted by RAB25 function and their link to pathogenic phenotypes in certain cancers. Furthermore, these data establish in an unbiased manner that RFP14 treatment antagonizes the RAB25-dependent gene expression program in a context where it also abrogates several RAB25-dependent phenotypes.

## Discussion

Despite the growing body of evidence supporting the role of RAB GTPases, and deregulated endocytic transport in general, in cancer, no cell-active probes have been developed targeting this class of proteins. Recent efforts to develop stabilized peptides targeting the RAB8a:RIP interaction yielded compounds with improved affinities in the micromolar range and protease resistance, but no reported activity in cells^32, 37^. Here we employed hydrocarbon peptide stapling, which has proven effective in targeting protein−protein interactions, to develop cell permeable, stabilized peptides capable of blocking RAB25-FIP complex formation. The data presented herein represent a detailed biochemical analysis of the FIP-RBDs from several FIP classes and their interactions with both RAB11a and RAB25. Importantly, we showed that RFP stapled peptides could be generated with high affinity for both isoforms, and that these compounds prevent RAB25-FIP complex assembly. The three lead compounds studied in cells, RFP14, RFP24 and RFP26, all exhibited suitable cell permeability as well as preferential binding to RAB25 over RAB11a. These are aspects of stapled peptide structure-function relationships that are difficult to design and assess a priori, and for this reason we sought to carry forward distinct active compounds. RFP14 exhibited preferential binding to RAB25 over RAB11a, and displayed RAB25-dependent effects in functional assays. Migration and proliferation assays established that RFP14 antagonized both loss- and gain-of-function phenotypes, as well as the global gene expression program regulated by RAB25. RFP26, on the other hand, exhibited RAB25-independent effects on the phenotypes explored here. As this compound was the most potent binder of RAB11a, these effects may stem from inhibition of RAB11a-mediated signaling or potentially other targets. The FIP-family proteins are primarily annotated as effectors of RAB11a/b and RAB25, and therefore the effects of RFP14 have been studied within this context here. However, further biochemical and cellular studies are warranted to explore how RFP14 and other optimized stapled peptides perturb the global RAB-GTPase network, crosstalk with other signaling pathways and whether these events could impact their effects on oncogenic phenotypes.

Here we report the first cellular-active probes of RAB25 signaling, and suggest that the RAB25-FIP interface can serve as a point of pharmacologic control over RAB25 signaling. We posit that the structure-activity relationships unearthed here will enable the design of future stapled peptides or peptidomimetics to target RAB11a and other RAB-family proteins (Supplementary Table 1). Finally, given the pro-tumorigenic role of RAB25 in several cancers, RFP14 represents a starting point for the development of therapeutics targeting RAB25 function in human cancers. Perhaps most important, we posit that these first-in-class chemical probes will enable, as evidenced historically with chemical probes for other protein classes^38^, future studies into RAB-family biochemistry and signal transduction both in normal and pathologic biological processes.

## Methods

### Cell lines and reagents

HEY, MCF7, MDA-MB231 cells lines were originally obtained from ATCC. The MDA-MB231 and MCF7 lines were transduced by Precision Lenti ORF, RAB25 construct or control (Thermo Scientific, Open Biosystem) followed by Blasticidin selection (30 μg/ml) up to 7 days. Subsequently limited serial dilution was performed and single cells were allowed to expand to separate colonies for genetically homogenous population. Two independent clones were obtained for each constructs and target gene expression was evaluated with western blotting. Additionally, a sequence confirmed hemagglutinin epitope-tagged *RAB25* (Invitrogen, Carlsbad, CA) expression vector on pcDNA 3.1 (referred to as pcDNA3) was used to generate stable HEY isogenic cell line.

Fmoc-protected natural amino acid precursors, activating reagents (HCTU) and solid phase support for chemical synthesis of stapled peptides were obtained from Novabiochem (EMD). Non-natural “*S*_5_” ((*S*)-N-Fmoc-2-(4′-pentenyl) alanine) amino acid was obtained from Anaspec Inc. Solvents and other chemical reagents were obtained from Sigma-Aldrich and used as received unless otherwise noted. HEY, MDA-MB231, OVCAR3 and MCF7 cells were from ATCC and were not STR profiled. Cell lines have been tested for mycoplasma contamination. HEY and MDA-MB231 cell lines were maintained in RPMI media supplemented with 10% fetal calf serum, 1% penicillin/streptomycin and 2 mM l-glutamine. OVCAR3 and MCF7 cell lines were maintained in RPMI-1640 media supplemented with 10% fetal calf serum and 1% penicillin/streptomycin.

### Stapled peptide synthesis and purification

Unmodified and hydrocarbon stapled peptides were synthesized by Fmoc-based solid phase peptide synthesis and purified by reverse-phase HPLC with a C18 column (Agilent, Palo Alto, CA) as previously reported^39^. The first series of RFP peptides (RFP1-RFP13) were analyzed by LC/MS using a C18 reverse-phase column (Agilent, 2.1 × 150 mm, pore size 80 Å, particle size 3.5 µM); Buffer A (H_2_O/0.1% TFA) and Buffer B (ACN/0.1% TFA); and a 15 min method with the following gradient (flow rate 0.5 mL/min): 10–100% buffer B over 10 min, 100% buffer B for 2 min, 100–10% buffer B over 1 min, and 10% buffer B over 2 min. Optimized RFP peptides (denoted by * on retention time in Supplementary Table 3) were analyzed by LC/MS using a C18 reverse-phase column (Phenomenex, 5.0 × 50 mm, pore size 110 Å, particle size 5 µm); Buffer A (5/95/0.1% ACN/H_2_O/TFA) and Buffer B (95:5:0.1% ACN/H_2_O/TFA); and a 20 min method with the following gradient (flow rate 0.5 mL/min): 0% buffer B over 3 min, 0–65% buffer B over 15 min, 65–100% buffer B over 1 min; 100–0% buffer B over 1 min. Purified peptides were lyophilized, quantified by A_280_, dissolved in DMSO as 10 mM stocks and stored at −20 °C.

### Recombinant protein constructs, expression, and purification

Recombinant RAB and FIP proteins were expressed in BL21 cells using the following vectors: GST-RAB25 (1–180) in pET41 Ek/LIC; His_6_-RAB25 (1–180) in pET28; GST-FIP3 (649–756) in pGEX 6P-1; GST-RAB11a (1–173, Q70L mutant) in pGEX5x-1. Transformed cells were incubated at 37 °C to an OD_600_ = 0.8, at which time induction was initiated with 1 mM IPTG for an additional 12 h at 20 °C. Cells were pelleted by centrifugation and lysed by sonication in lysis buffer (20 mM Tris pH 8.0, containing 150 mM NaCl, 5 mM MgCl_2_, 2 mM DTT, 2% glycerol and protease inhibitors (EDTA-free, Roche)). GST-tagged proteins were purified by binding to glutathione 4B sepharose resin (GE Lifesciences) at 4 °C, washed three times in lysis buffer, followed by elution with 20 mM reduced glutathione in lysis buffer. His-tagged RAB25 was expressed and lysed as above, prior to loading onto Ni-NTA resin (Qiagen), washed with lysis buffer containing 10 mM imidazole and eluted in lysis buffer containing 250 mM imidazole. All proteins were further purified by FPLC gel filtration (Superdex 75, GE Lifesciences) and dialyzed into appropriate assay buffers for downstream analysis.

### Fluorescence polarization assays

Fluorescence polarization assays were performed with for GST-RAB25 and GST-RAB11a to quantify binding to FITC-labeled unmodified and stapled RFP peptides. Initial fluorescence polarization measurements for RFP1−RFP13 were made by incubating FITC-RFP peptides (25 nM) with threefold dilutions of a given protein in FP buffer, consisting of 20 mM Tris pH 8.0, 150 mM NaCl, 5 mM MgCl_2_, 2 mM DTT, 10 μM GTP and 2% glycerol. Dilutions and incubations were performed in 96-well, black flat-bottom plates (Nunc) to a total volume of 100 μL, and equilibrated at room temperature for 30 min. Polarization was measured on a Spetramax-M5 multi-label plate reader (Molecular Devices) with *λ*_ex_ = 485 nm and *λ*_em_ = 525 nm. Polarization values were determined using the equation: *P* = (*V − H*)/(*V + H*), where *P* denotes polarization, *V* denotes vertical emission intensity and *H* represents horizontal emission intensity. Fraction bound was calculated using the equilibrium maximum polarization value obtained for a given protein among ligands, to which background-subtracted fluorescence polarization values were internally normalized. Fluorescence polarization measurements for FITC-RFP14, 24, 26, 31, and 32 were performed as detailed above with the exception that 10 nM FITC-ligand was used. Binding curves, apparent *K*_d_ values and 95% confidence intervals were determined generated using Prism 5 graphing software by fitting data to sigmoidal binding curve (4-parameter) according to the equation:

*Y* = bottom + (top−bottom)/(1 + 10^(LogEC^_50_^ – X)×HillSlope^) as detailed on the Prism 5 website.

### ALPHAscreen assays

ALPHAscreen assays were performed using alpha buffer, consisting of FP Buffer supplemented with 0.1% BSA (w/v) in white, flat-bottom 384-well ALPHAscreen plates (Perkin Elmer). For bimolecular association assays in Fig. 2b, biotinylated RFP peptide and tagged RAB25 protein was diluted to 2× the indicated final concentrations in 20 μL of buffer on the ALPHAscreen plates and incubated for 30 min at room temperature. Following incubation, 10 μL of 4× ALPHAscreen donor beads (10 μg/mL final concentration, Streptavidin-linked, Perkin Elmer) was added and incubated for 15 min at room temperature prior to final addition of 10 μL of 4× ALPHAscreen acceptor beads (Ni^2+^-NTA-linked or anti-GST, 10 μg/mL final concentration, Perkin Elmer) and incubated for an additional 30 min. Proximity-based luminescence was measured on an ALPHAscreen-capable EnVision plate reader (Perkin Elmer) according to the manufacturers settings and protocols. Peptide competition assays in Fig. 3c were performed as above with the indicated concentrations of soluble peptide or proteins incubated with biotin-RFP14 and H_6_-RAB25 (0.2 μM each in all wells) prior to addition of donor and acceptor beads. Binding curves, IC_50_ values and 95% confidence intervals were determined generated using Prism 5 graphing software by fitting data to sigmoidal inhibition curves (4-parameter) according to the equation: *Y* = bottom + (top−bottom)/(1 + 10^(LogIC^_50_^ – X)×HillSlope^) as detailed on the Prism 5 website.

### Circular dichroism spectroscopy

Circular dichroism spectroscopy experiments were performed on a Jasco J-170 using a quartz cuvette (path length: 0.1 cm). Peptides were dissolved to 50 μM in deionized water and CD measurements were recorded at one nm increments between 190 and 260 nm, at room temperature. Thermal denaturation experiments were performed by recording CD absorbance at 222 nm at one-degree increments from 10 to 90 °C using a thermostat-controlled cuvette chamber. Raw denaturation data were fit to sigmoidal curves with Prizm 5 software, and the half-maximal temperature was reported as the peptide *T*_m_.

### Cellular pull-down assays and western blotting

HEY-pcDNA and HEY HA-RAB25 cells were transfected with pcDNA3-His B-FIP1 (kind gift from Dr. Jim Norman)^40^ using Lipofectamine 2000 transfection reagent following manufacturers’ protocol (Invitrogen). Forty-eight hours post transfection, the cells were collected using the HA-Lysis buffer provided in the Sigma HA-IP kit (IP0010-1KT Sigma-Aldrich, St. Louis, MO**)** and pretreated with either DMSO or indicated concentrations of the stapled RFP compounds for 1 h at 4 °C. Subsequently, ~600 μg of total protein was loaded in each IP column subjected to immunoprecipitation using agarose beads coupled to anti-HA monoclonal antibody. Unbound proteins were removed by extensive washing and specifically associated proteins resolved by SDS-PAGE and western blotting for detection of RAB25-FIP1 interaction under various treatment conditions. Normal IgG was used as negative control.

Western blotting was performed using primary antibodies for RAB25 (Cell Signaling 4314; 1:500), RAB11a (Cell Signaling 2413; 1:1000) and FIP1 (Gift from Dr Jim Norman, 1:1000)^40^, GAPDH (Santa Cruz; 1:1000), and secondary antibodies, anti-rabbit or anti-mouse immunoglobulin G (IgG) horseradish peroxidase-linked secondary antibody (Cell Signaling Technology; 1:2000). Full western blots are presented in Supplementary Fig. 4.

### Confocal fluorescent microscopy for peptide cellular penetration

MCF7 cells were grown in 8-well chamber slides (Ibidi) and treated with FITC-labeled RFPs (5 μM) in 10% FBS-containing RPMI for 8 h at 37 °C. Cells were washed 5× in PBS, fixed in 2% paraformaldehyde/PBS and stained with DAPI according to the manufacturer’s protocol (Sigma). A coverslip was mounted onto the slide and confocal fluorescence microscopy performed with an Olympus DSU spinning disk confocal microscope (Olympus Corporation of the Americas, Center Valley, PA) with a Hamamatsu model C9100 EM-CCD camera (Hamamatsu Photonics, Skokie, IL) run by SlideBook v5.0 software (Intelligent Imaging Innovations, Denver, CO). Post-acquisition processing (multi-channel overlay) was performed using ImageJ software (NIH). Results are representative of images taken from three fields across the same well in at least two biological replicates.

### Cell proliferation studies

The day before treatment, control and stable RAB25 overexpressing HEY, MDA-MB231, and MCF7 cells (8 × 10^3^ cells) were seeded in 96-well plates in culture medium containing 5% FBS overnight. The next day cells were washed twice with PBS and treated with DMSO or RFP peptides (15 μM) in 0.5% FBS containing medium for 48 h. Cell viability was determined using 8% PrestoBlue (Life Technologies, Federick MD), a resazurin-based solution that functions as a cell viability indicator, which was read (excitation wavelength 530 nm; emission wavelength 604 nm) using a TECAN microplate reader at 0 h and 48 h. For untreated cells, the reagent was added at 24, 48, and 72 h post plating (in 0.5% FBS containing RPMI medium) and absorbance was recorded at each timepoint.

### Cell migration assays

Cells were seeded at a density of 5000–7000 cells for HEY and MDA-MB231 or 15,000 cells for MCF7 on each side of an Ibidi culture insert (Ibidi, Munich, Germany), with a 500 μm separation between each side of the well, and allowed to grow for 24 h to attain a compact monolayer. Cells were pretreated with, or without, RFP peptides (10 μM) or DMSO control for 12 h before removal of the insert, and following removal of the insert cells were incubated in RFP-containing media or DMSO control for the duration of wound closure. Cells were photographed using a Life Technologies EVOS XL Core imaging system 4× or a Nikon DS camera connected to a Nikon Eclipse Ti microscope objective at insert removal (0 h) and when wound closure was complete under control conditions. Experiments were performed in duplicate and a minimum of four fields of the injury were photographed and used for quantification.

### Cellular thermal shift assay

Following sonication and centrifugation of MCF7 cells in Mammalian RAB lysis buffer (20 mM Tris pH 8.0, 150 mM NaCl, 5 mM MgCl_2_, 2 mM DTT, 0.1% TritonX-100, and protease inhibitors (EDTA-free, Roche)), bulk samples of lysate (diluted to 1 mg/mL) were incubated with 10 µM of the indicated peptide (or DMSO) for 10 min. Lysates were then aliquoted into 200 µL PCR tubes, heated for 3 min at a temperature point between 37 and 70 °C, incubated an additional 3 min at room temperature, centrifuged (15,000×*g* for 20 min), and supernatants were removed and combined with SDS-PAGE loading buffer for western blot analysis, and then analyzed by western blot (blotting for either RAB25, 11a, or GAPDH as negative control) and downstream ImageJ quantification. A sigmoidal curve was fit to the resulting data from each target protein for each of the three sets of treatment conditions, from which *T*_m_ values were calculated.

### Gene expression profiling

*RNA isolation cDNA, library preparation and capture.* For the conditions listed, RAB25-HEY cells (10 cm plate, 70–80% confluent) were treated with RFP14 (10 μM) or the equivalent amount of DMSO in serum free media overnight, followed by stimulation with 5% FBS for 8 h. pcDNA3-HEY cells were treated with DMSO overnight and stimulated with 5% FBS for 8 h in parallel. Individual biological replicates performed on different days were used for RNAseq studies. Samples were snap frozen in liquid nitrogen at the time of collection and total RNA was isolated from each condition by Norgen Total RNA Purification Kit (Norgen Biotek Corp, ON Canada), quantified by Picogreen (Invitrogen), and quality was assessed using a 2200 Tapestation (Agilent). RNA from each biological replicate (500 ng) was converted to double-stranded cDNA using Ovation RNA-Seq System V2 kit (Nugen), and cDNA was sheared by sonication with the following conditions: Peak Incident Power = 175, Duty Cycle = 20%, Intensity = 5, Cycles per burst = 200, for a time = 120 s using Covaris E220 sonicator (Covaris). To ensure the proper fragment size, samples were checked on a TapeStation using the DNA High Sensitivity kit (Agilent). The sheared DNA proceeded to library preparation using KAPA library prep hyper kit (KAPA) following the “with beads” manufacturer protocol. Briefly, this protocol consists of three enzymatic reactions for end repair, A-tailing and adaptor ligation, followed by barcode insertion by PCR using KAPA HiFi polymerase (6 cycles). PCR primers were removed by using 1.8× volume of Agencourt AMPure PCR Purification kit (Agencourt Bioscience Corporation). At the end of library preparation, samples were analyzed by TapeStation to verify correct fragment size and the absence of extra bands. Samples were quantified using KAPA qPCR quantification kit. Equimolar amounts of DNA were pooled for capture (2–6 samples per pool). We used whole exome biotin labeled probes from Roche Nimblegen (V3) and followed manufacture’s protocol for the capture step. Briefly, DNA was pooled (2–6 samples), dried, resuspended with capture reagents and probes, and incubated at 47 °C on a thermocycler with a heated lid (57 °C) for 64–74 h. Targeted regions were recovered using streptavidin beads, streptavidin-biotin-probe-target complexes were washed, and another round of PCR amplification was performed according to manufacturer’s protocol. The quality of each captured sample was analyzed by TapeStation using the DNA High Sensitivity kit, and the enrichment was accessed by qPCR using specific primers designed by Roche Nimblegen. The minimum cutoff for the enrichment was 50-fold.

### RNA sequencing and data analysis

The captured libraries were sequenced on a HiSeq 2000 (Illumina Inc., San Diego, CA, USA) on a version 3 TruSeq paired-end flowcell at a cluster density between 700 and 1000 K clusters/mm^2^ according to manufacturer’s instructions. Sequencing was performed on a HiSeq 2000 for 2 × 100 paired-end reads with a 7 nt read for indexes using Cycle Sequencing v3 reagents (Illumina). The resulting BCL files containing the sequence data were converted into “.fastq.gz” files and individual libraries within the samples were demultiplexed using CASAVA 1.8.2 with no mismatches. All regions were covered by > 20 reads. Data analysis was performed using a comprehensive in-house RNA-Seq pipeline (IPCT, Bioinformatics and Computational Biology, M.D. Anderson). STAR was used to align paired-end reads to the hg19 version of the reference genome, featureCount was used to obtain expression counts of genes and exons, and Cufflinks was used to estimate gene expression (FPKM). Genetic variants were called using GATK unified genotyper. Fusions were detected using STAR and filtered using Oncofuse. Quality of raw and aligned reads was assessed using FastQC and Qualimap.

FPKM values of six technical samples (pcDNA3-HEY + DMSO, RAB25-HEY + DMSO, RAB25-HEY + RFP14, each from individual biological replicates) were normalized according to total FPKM values of 34,560 genes. Genes with zero expression in one or more samples compared to FPKM values of the rest of the 22,912 genes were eliminated. To define the set of transcripts that were consistently and differentially expressed as a result of RAB25 expression, a mean fold-change of > 2.14 was applied (to control the number of genes between 100 and 500, which is optimal for GSEA analysis) between pcDNA-HEY and RAB25-HEY RNAseq replicates. This resulted in gene sets of 104 transcripts that were upregulated by RAB25 (named RAB25_UP) and 269 transcripts that were downregulated by RAB25 (named RAB25_DOWN). Gene set enrichment analysis (GSEA) was performed using the GSEA portal (http://www.broad.mit.edu/GSEA/) in the following way. Each gene set was queried for enrichment within the expression profile generated by comparing DMSO and RFP14 treated RAB25-HEY cells, thereby asking whether RAB25-regulated genes are specifically regulated by RFP14 in an unbiased manner. GSEA was performed with the following parameters: probe set collapse = false; phenotype = RFP14 vs. DMSO; permutation: sample, permutations = 1000. Gene set size: 15 < *n* < 500. Heat maps showing the per-gene effect of RFP14 on the RAB25-regulated genes (each in the RAB25_UP and RAB25_DOWN gene sets) were generated using Cluster 3.0, and visualized using Tree View software. Samples are plotted in columns and the genes in rows.

### qRT-PCR and statistical analysis

HEY ovarian cancer cells with stable ectopic expression of RAB25 were plated and allowed to reach 70% confluency before treating with DMSO, RFP14, or RFP32 (10 μM or equivalent DMSO) in serum free media for 12 h, followed by stimulation with 5% FBS for 8 h. Total cellular RNA was then isolated from cells using Thermo RNA Isolation kit (Thermo Fisher Scientific, MA, Cat #AM1560), according to the manufacturer’s instructions. Two micrograms of RNA per sample was reverse transcribed to generate complementary DNA, using reverse transcriptase Thermo RT kit (Cat #4368814). qRT-PCR was performed using a 7500 Fast Real-time PCR System (Applied Biosystems Inc., CA) with SYBR Green Power master mix (Thermo Cat #4309155). The primers used for the *ACTB* and *18 S* (internal control) and other listed genes were designed using the ‘Primer 3 Output’ software from cDNA sequences found in the NCBI Gene Database (Nucleotide). Their specificity was confirmed using a BLAST analysis against the genomic NCBI database. qRT-PCR reaction conditions were: 50  °C for 2 min; 95  °C for 2 min; 40 × (95  °C for 15 s; 60  °C for 15 s; 72  °C for 1 min). The relative quantification of gene expression between the treatments and control cells was calculated by the 2^−ΔΔCt^ approximation method. Primers used for qPCR analysis are listed below.

### Statistics statements

All experiments consisted of triplicate measurements, with biological or technical replicates indicated. All center values given refer the mean and error bars shown represent the standard error of the mean, unless otherwise stated. Sigmoidal binding curves were applied using Prism software and affinites or IC_50_ values reported represent the mean and the 95% confidence interval. Asterisks in figure legends refer to *P*-value thresholds of < 0.05 (*), < 0.01 (**), or < 0.005 (***) from two-sided Student’s *t*-test. No statistical methods or power calculations were used to for sample size determination.

### Data availability

The RNAseq data that support the findings in this study are available in the National Center for Biotechnology Information Gene Expression Omnibus (GEO) and are accessible through accession number GSE101528.

## Electronic supplementary material

Supplementary Information
