# Supplementary material for: Stapled peptide inhibitors of RAB25 target context-specific phenotypes in cancer
Source: Nat Commun. 2017 Sep 22;8:660. doi: 10.1038/s41467-017-00888-8 (PMC5610242; doi:10.1038/s41467-017-00888-8)
Supplement: Supplementary file 1 — Supplementary Information [file 41467_2017_888_MOESM1_ESM.pdf]

Description of Supplementary Files

File Name: Supplementary Information
Description: Supplementary Figures and Supplementary Tables

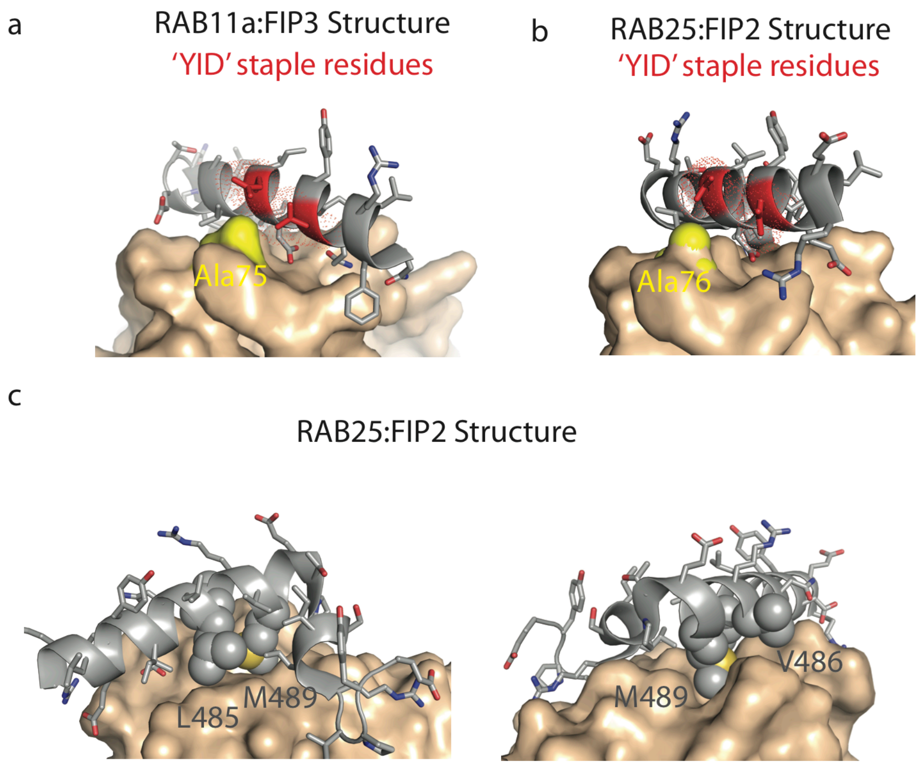

Supplementary Figure 1. Relevant interactions highlighted in RAB11a:FIP3 and RAB25:FIP2 crystal structures. **a-b**) Potential steric clash between the hydrocarbon staple in peptides harboring the 'YID' staple position with Ala75 or Ala76 in RAB11a and RAB25, respectively. **c**) The hydrophobic residues L485, V486 and M489 in FIP2 make critical contacts with the RAB25 binding interface and are the sites of alanine substitution in the negative control peptides RFP31 and RFP32. RAB11a:FIP3 PDB accession: 2HV8; RAB25:FIP2 PDB accession: 3TSO.

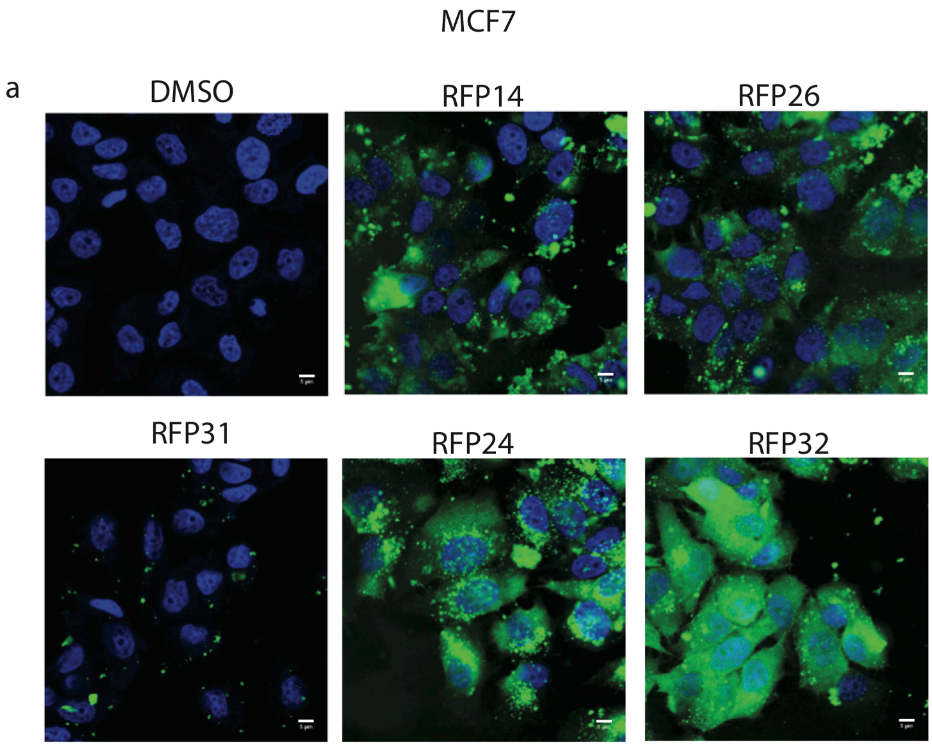

Supplementary Figure 2.

FITC-RFP peptides are cell permeable. **a**) Confocal fluorescence imaging of DAPI-stained MCF7 cells treated with FITC-RFP peptides in 10% FBS-containing RPMI (5 μM, 8 hr). FITC-RFP14, 24, and 26 show comparable cellular uptake of peptide, whereas one negative control appears less permeable (FITC-RFP31) and the other (FITC-RFP32) appears slightly more permeable. DMSO-treated control cells show no background fluorescence under the acquisition settings. Inset white scale bars = 5 μm.

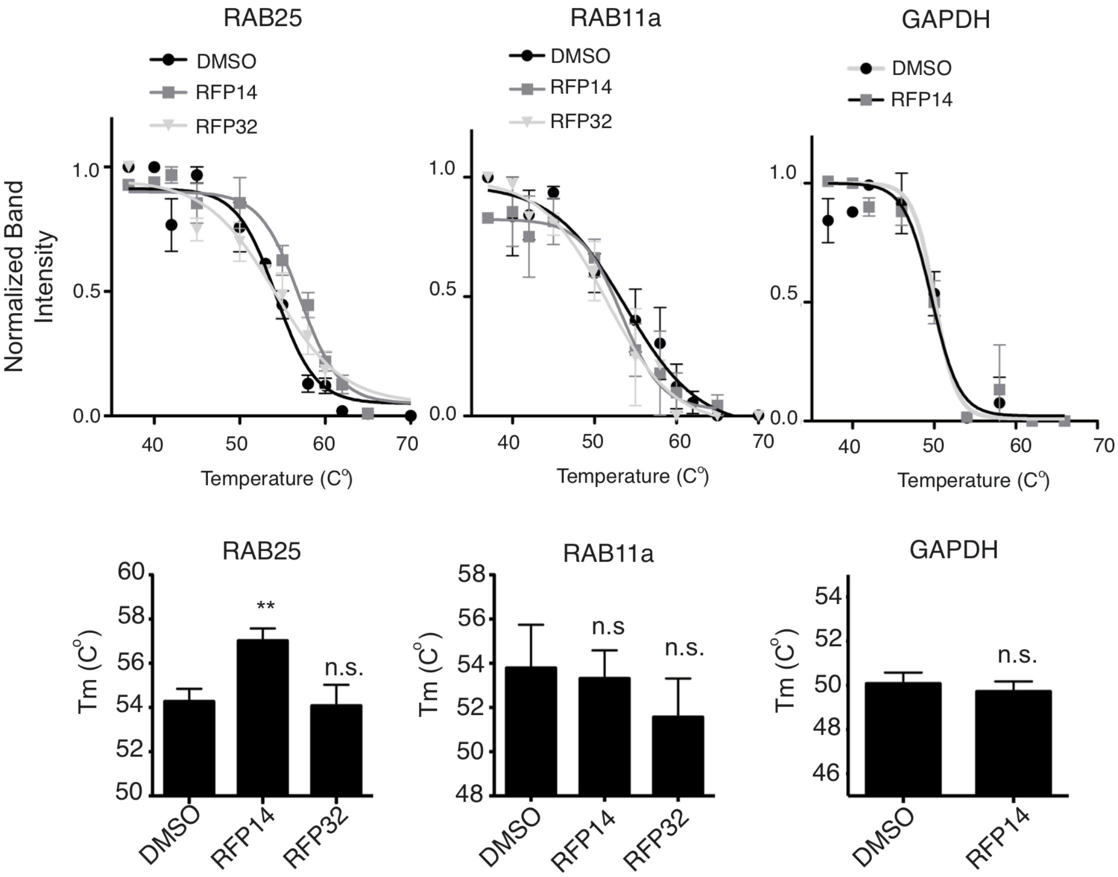

Supplementary Figure 3. Cellular thermal shift assay denaturation curves (*top*) and Tm values (*bottom*) from indicated treatments in MCF7 cells. Lysate samples were incubated with the indicated peptide at 10 µM for 30 min, aliquoted, and processed for CETSA analysis and western blot. The only significant and positive Tm-shift is seen for RAB25 with RFP14 treatment, which is indicative of target-binding and thermal-stabilization. These results are representative of at least two technical replicates from two or more biological replicates. Data points represent the mean ± s.e.m. from three or more technical replicates. Tm values represent mean with s.e.m from the application of a sigmoidal curve fit using Prism 5 software.

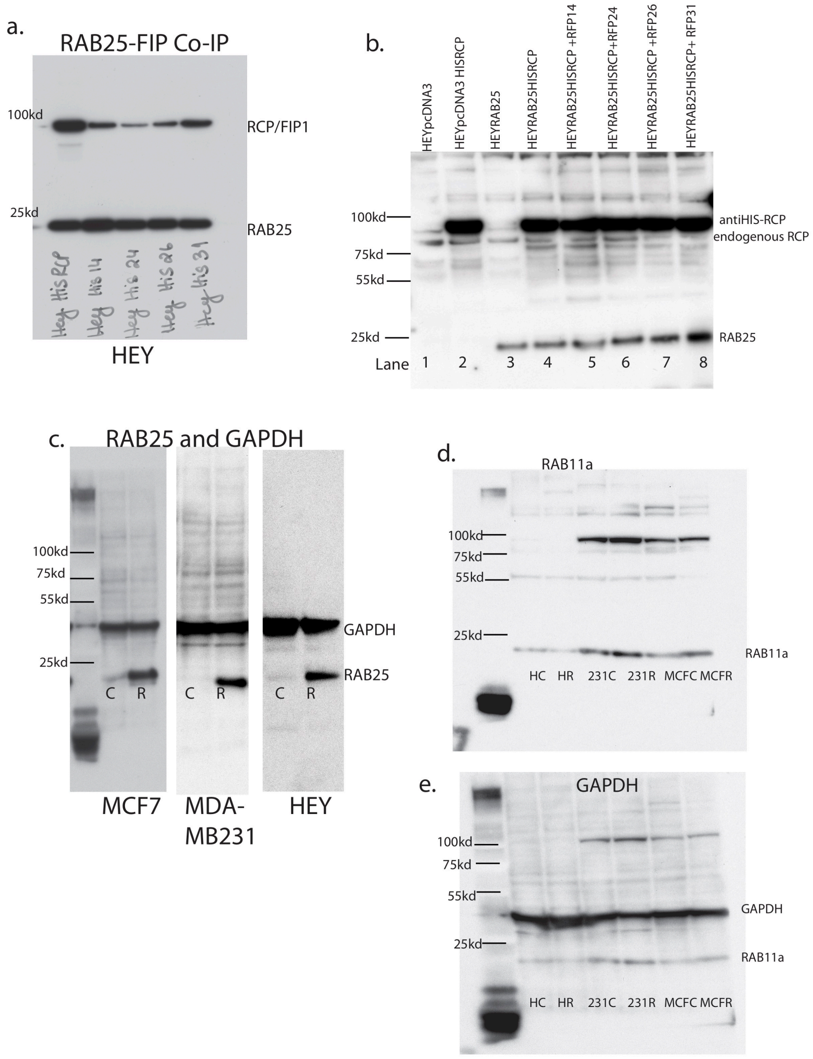

Supplementary Figure 4

Representative full gels from western blot experiments for the following figures: **a**) Full blot of co-immunoprecipitation following RFP treatments in Fig. 3d; **b**) Whole cell lysate input used for IP (lanes 4-8) in Fig. 3d and with additional HEYpcDNA3cont and HEY-RAB25 cells with or without His-RCP/FIP1 expression. **c)** Fig. 4a, showing RAB25 and GAPDH levels in isogenic lines. **d**) Whole blot in Fig. 4b staining endogenous RAB11a levels. **e**) GAPDH loading control for Fig. 4b. Lanes are marked C= cont, R= RAB25

overexpression, H=HEY, 231= MDA-MB231, MCF=MCF7. These blots depict the raw signal without any data normalization performed on them.

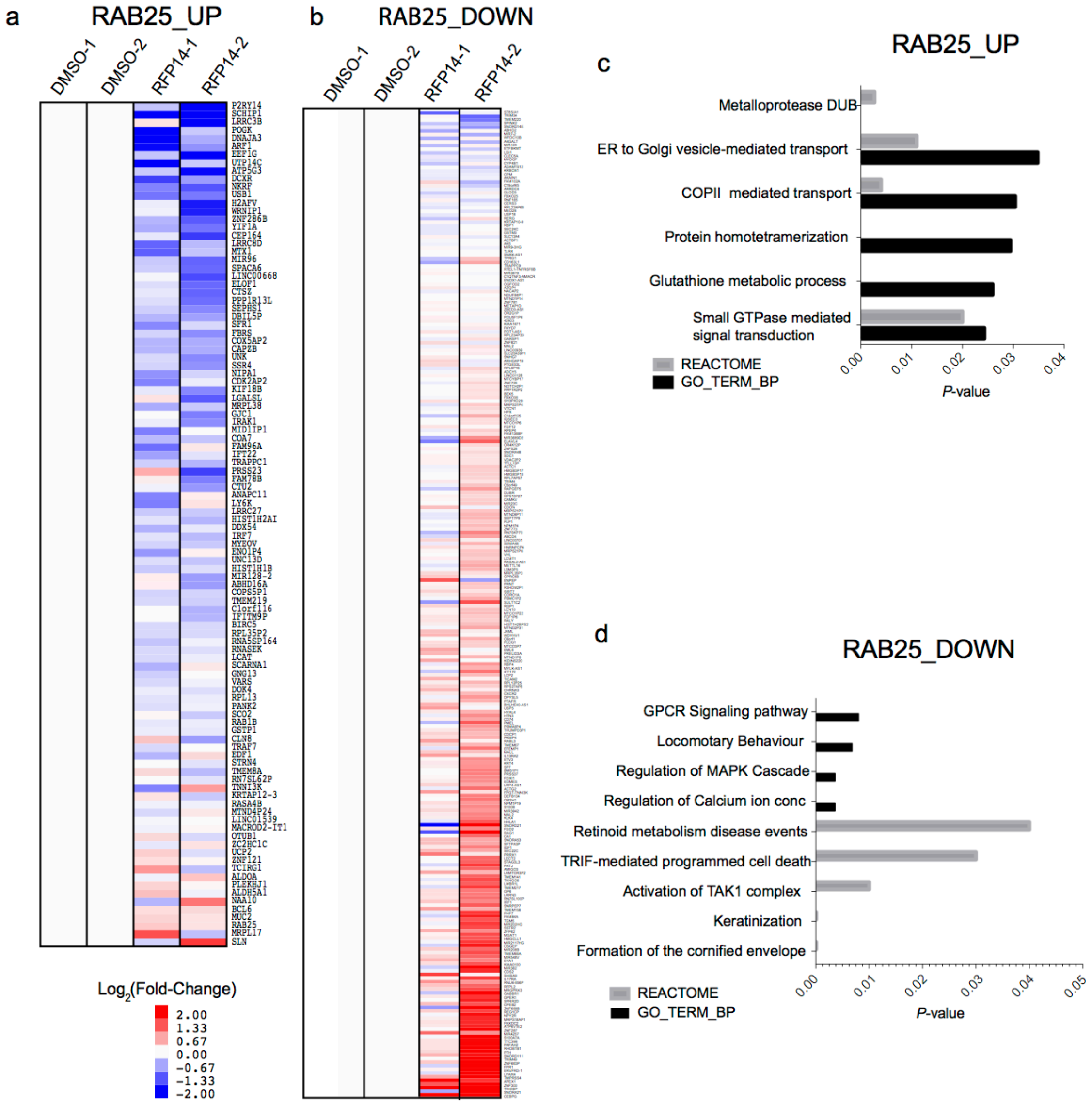

Supplementary Figure 5. RFP14 opposes the global RAB25-dependent gene expression program in HEY ovarian cancer cells. **a,b**) Heat map representations showing the effect of RFP14, normalized on a per-gene basis to DMSO, on the expression of each gene in the RAB25_UP (a) and RAB25_DOWN gene sets in RAB25-expressing HEY cells. Heat maps are derived from the GSEA analyses presented in Figure 6 of the main text. **c, d**) Gene ontology biological process categories (GOTERM_BP, identified by DAVID

bioinformatic analysis) and REACTOME pathways that were significantly enriched in the RAB25_UP (c) and RAB25_DOWN gene sets (d) are plotted according to enrichment *P*-value.

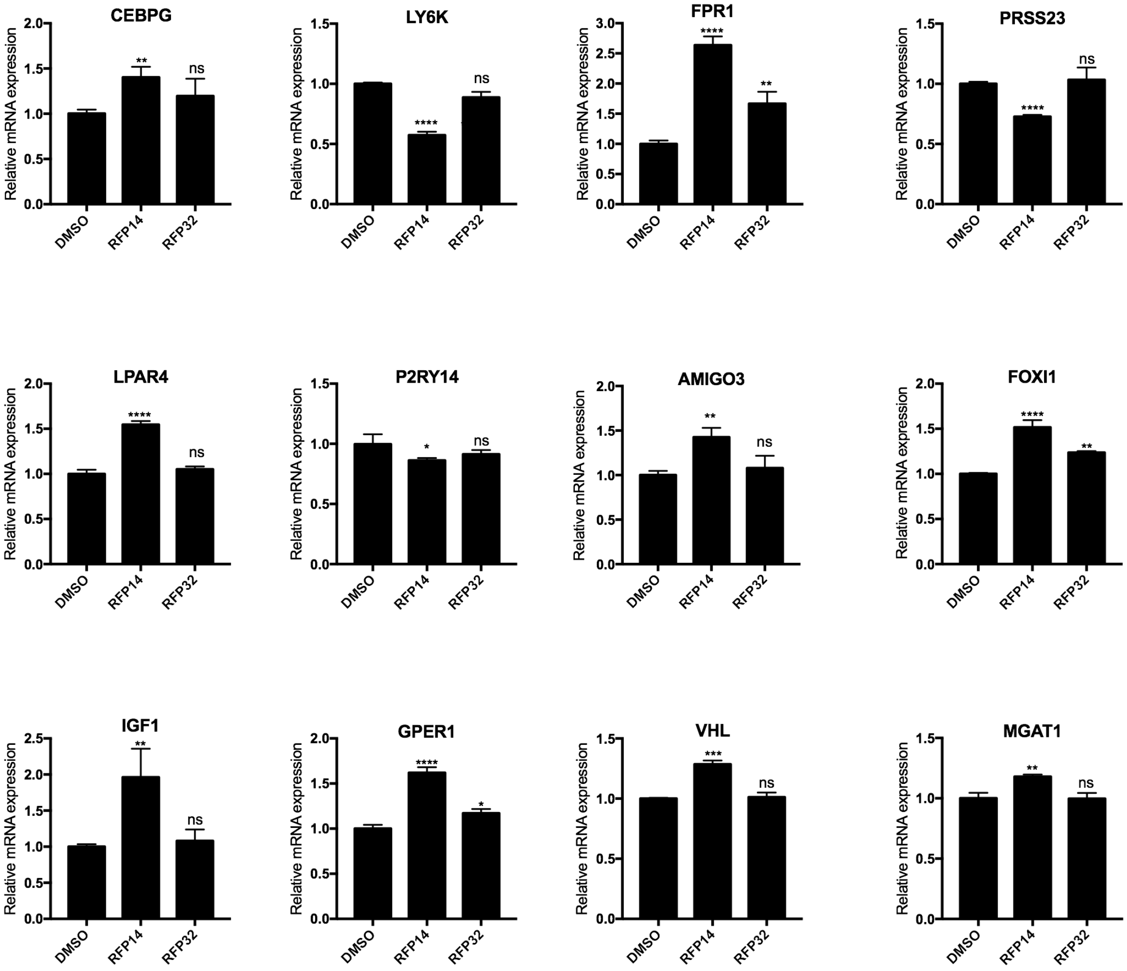

Supplemental Figure 6. Quantitative real-time PCR gene expression analysis of RAB25-dependent genes in response to DMSO, RFP14 or RFP32 treatment (10 µM, 8 hr) of RAB25-expressing HEY cells. Mean expression values are shown as relative changes to DMSO treatment alone, and all genes are normalized by *ACTB*. Shown are triplicate technical replicate measurements from triplicate biological experiments, with the bars indicated mean +/- s.d.. **P* < 0.05; ***P*< 0.01; ****P*< 0.001, Students *t*-test. N.S, not significant.

Peptide	Sequence	Apparent K_D, (95% C.I.) / (μM) RAB11a	RAB25	T_m at 222 nm	Cell Uptake	Inhibition of RAB25-dependent phenotypes
RFP1	βQVRELEDYIDNLLVRVMEETPNILRIP	>5	>5			
RFP2	βHIRELEDYIDNLLVRVMEETPSILRVP	>5	>5			
RFP3	βNFRLQDYIDRIIVAIMETNPSILEVK	2.3 (1.6-3.4)	>5			
RFP4	βNFRLRQYMDKIILAILDHNPSILEIK	0.93 (0.4-2.2)	2.3 (1.3-4.3)	37	-	-
RFP5	βNFRLQDYIDRIIVAIM_{ox}ETNPSILEVK	>5	>5			
RFP6	βRQVRELES_5YIDS_5LLVRVMEETPNILRIPR	>5	>5			
RFP7	βRQVRELEDYIDS_5LLVS_5VMEETPNILRIPR	0.11 (0.07-0.17)	0.51 (0.41-0.65)			
RFP8	βRQVRELEDYIDRLLVS_5VMES_5TPNILRIPR	0.04 (0.03-0.05)	0.17 (0.08-0.37)			
RFP9	βRQVRELEDS_5IDRS_5LVRVMEETPNILRIPR	2.4 (1.4-4.0)	1.2 (0.80-1.8)			
RFP10	βRINFRLQS_5YIDS_5IIVAIMETNPSILEVK	>5	>5			
RFP11	βRINFRLQDYIDS_5IIVS_5IMETNPSILEVK	0.007 (0.005-0.01)	0.05 (0.03-0.08)			
RFP12	βRINFRLQDYIDRIIVS_5IMES_5NPSILEVK	0.02 (0.007-0.06)	0.03 (0.02-0.06)			
RFP13	βRINFRLQDS_5IDRS_5IVAIMETNPSILEVK	0.08 (0.05-0.12)	0.04 (0.02-0.10)			
RFP14	βRQVRELENYIDRLLVS_5VN_LES_5TPNILRIPR	0.32 (0.21-0.49)	0.04 (0.025-0.078)	60	++	+
RFP24	βRINFRLQNS_5IDRS_5IVRIFETNPSILRVK	0.56 (0.44-0.74)	0.16 (0.11-0.23)	52	++	+
RFP26	βRINFRLQNS_5IDRS_5IVRIN_LETNPSILRVK	0.09 (0.064-0.12)	0.02 (0.016-0.029)	69	++	-
RFP31	βRINFRLQNS_5IDRS_5AARIAETNPSILEVK	>5	>5	59	+	-
RFP32	βRINFRLQNS_5IDRS_5AARIAETNPSILRVK	>5	>5	54	++	-

Supplementary Table 1. Stapled RFP peptide structure activity relationships. Name, sequence, binding affinities to RAB11a and RAB25, structural characteristics and cell-based data for the unmodified and stapled RFP peptides analyzed in this study. All blank positions represent assays that were not performed. Negative (-), positive (+) and double positive (++) signs are qualitative indicators of compound performance in an indicated assay. Relevant biochemical, structural and cell-based parameters for the main compounds utilized in this study are presented here for structure activity relationship analysis; additional quantitative annotations for the given compounds are presented in the main text.

PROBE	NAME	CHANGE	PROBE	NAME	CHANGE
ABHD16A	abhydrolase domain containing 16A(ABHD16A)	UP	MID1IP1	MID1 interacting protein 1(MID1IP1)	UP
ALDH5A1	aldehyde dehydrogenase 5 family member A1(ALDH5A1)	UP	MIR128-2	microRNA 128-2(MIR128-2)	UP
ALDOA	aldolase, fructose-bisphosphate A(ALDOA)	UP	MIR96	microRNA 96(MIR96)	UP
ANAPC11	anaphase promoting complex subunit 11(ANAPC11)	UP	MRPL17	mitochondrial ribosomal protein L17(MRPL17)	UP
ARF1	ADP ribosylation factor 1(ARF1)	UP	MRPL38	mitochondrial ribosomal protein L38(MRPL38)	UP
ATP5G3	ATP synthase, H+ transporting, mitochondrial Fo complex subunit C3 (subunit 9)(ATP5G3)	UP	MTND4P24	mitochondrially encoded NADH:ubiquinone oxidoreductase core subunit 4 pseudogene 24(MTND4P24)	UP
BCL6	B-cell CLL/lymphoma 6(BCL6)	UP	MTX1	metaxin 1(MTX1)	UP
BIRC5	baculoviral IAP repeat containing 5(BIRC5)	UP	MUC2	mucin 2, oligomeric mucus/gel-forming(MUC2)	UP
C1orf116	chromosome 1 open reading frame 116(C1orf116)	UP	MYEOV	myeloma overexpressed(MYEOV)	UP
CAPZB	capping actin protein of muscle Z-line beta subunit(CAPZB)	UP	NAA10	N(alpha)-acetyltransferase 10, NatA catalytic subunit(NAA10)	UP
CDK2AP2	cyclin dependent kinase 2 associated protein 2(CDK2AP2)	UP	NIPA1	non imprinted in Prader-Willi/Angelman syndrome 1(NIPA1)	UP
CEP164	centrosomal protein 164(CEP164)	UP	NKRF	NFKB repressing factor(NKRF)	UP
CLN8	ceroid-lipofuscinosis, neuronal 8(CLN8)	UP	OTUB1	OTU deubiquitinase, ubiquitin aldehyde binding 1(OTUB1)	UP
COA7	cytochrome c oxidase assembly factor 7 (putative)(COA7)	UP	P2RY14	purinergic receptor P2Y14(P2RY14)	UP
COPS5P1	COP9 signalosome subunit 5 pseudogene 1(COPS5P1)	UP	PANK2	pantothenate kinase 2(PANK2)	UP
COX5AP2	cytochrome c oxidase subunit 5A pseudogene 2(COX5AP2)	UP	PLEKHJ1	pleckstrin homology domain containing J1(PLEKHJ1)	UP
CTSZ	cathepsin Z(CTSZ)	UP	POGK	pogo transposable element with KRAB domain(POGK)	UP
CTU2	cytosolic thiouridylase subunit 2(CTU2)	UP	PPP1R13L	protein phosphatase 1 regulatory subunit 13 like(PPP1R13L)	UP
DBIL5P	diazepam binding inhibitor-like 5, pseudogene(DBIL5P)	UP	PRSS23	protease, serine 23(PRSS23)	UP
DCXR	dicarbonyl and L-xylulose reductase(DCXR)	UP	RAB1B	RAB1B, member RAS oncogene family(RAB1B)	UP
DDX54	DEAD-box helicase 54(DDX54)	UP	RAB25	RAB25, member RAS oncogene family(RAB25)	UP
DNAJA3	DnaJ heat shock protein family (Hsp40) member A3(DNAJA3)	UP	RASA4B	RAS p21 protein activator 4B(RASA4B)	UP
DOK4	docking protein 4(DOK4)	UP	RN7SL62P	RNA, 7SL, cytoplasmic 62, pseudogene(RN7SL62P)	UP
EDF1	endothelial differentiation related factor 1(EDF1)	UP	RNA5SP164	RNA, 5S ribosomal pseudogene 164(RNA5SP164)	UP
EEF1G	eukaryotic translation elongation factor 1 gamma(EEF1G)	UP	RNASEK	ribonuclease K(RNASEK)	UP
ELOF1	elongation factor 1 homolog(ELOF1)	UP	RPL13	ribosomal protein L13(RPL13)	UP
ENO1P4	enolase 1 pseudogene 4(ENO1P4)	UP	RPL35P2	ribosomal protein L35 pseudogene 2(RPL35P2)	UP
FAM78B	family with sequence similarity 78 member B(FAM78B)	UP	SCARNA1	small Cajal body-specific RNA 1(SCARNA1)	UP
FAM96A	family with sequence similarity 96 member A(FAM96A)	UP	SCHIP1	schwannomin interacting protein 1(SCHIP1)	UP
FBRS	fibrosin(FBRS)	UP	SCO2	SCO2, cytochrome c oxidase assembly protein(SCO2)	UP
GJC1	gap junction protein gamma 1(GJC1)	UP	SEPHS1	selenophosphate synthetase 1(SEPHS1)	UP
GNG13	G protein subunit gamma 13(GNG13)	UP	SFR1	SWI5 dependent homologous recombination repair protein 1(SFR1)	UP
GSTP1	glutathione S-transferase pi 1(GSTP1)	UP	SLN	sarcolipin(SLN)	UP
H2AFV	H2A histone family member V(H2AFV)	UP	SPACA6	sperm acrosome associated 6(SPACA6)	UP
HIST1H1B	histone cluster 1 H1 family member b(HIST1H1B)	UP	SSR4	signal sequence receptor subunit 4(SSR4)	UP
HIST1H2AI	histone cluster 1 H2A family member i(HIST1H2AI)	UP	STRN4	striatin 4(STRN4)	UP
IFITM9P	interferon induced transmembrane protein 9 pseudogene(IFITM9P)	UP	TCIRG1	T-cell immune regulator 1, ATPase H+ transporting V0 subunit a3(TCIRG1)	UP
IFT22	intraflagellar transport 22(IFT22)	UP	TMEM219	transmembrane protein 219(TMEM219)	UP
IRAK1	interleukin 1 receptor associated kinase 1(IRAK1)	UP	TMEM8A	transmembrane protein 8A(TMEM8A)	UP
IRF7	interferon regulatory factor 7(IRF7)	UP	TNNI3K	TNNI3 interacting kinase(TNNI3K)	UP
KIF18B	kinesin family member 18B(KIF18B)	UP	TRAF7	TNF receptor associated factor 7(TRAF7)	UP
KRTAP12-3	keratin associated protein 12-3(KRTAP12-3)	UP	TRAPPC1	trafficking protein particle complex 1(TRAPPC1)	UP
LCAT	lecithin-cholesterol acyltransferase(LCAT)	UP	UCP2	uncoupling protein 2(UCP2)	UP
LGALSL	galectin like(LGALSL)	UP	UNC13D	unc-13 homolog D(UNC13D)	UP
LINC00668	long intergenic non-protein coding RNA 668(LINC00668)	UP	UNK	unkempt family zinc finger(UNK)	UP
LINC01539	long intergenic non-protein coding RNA 1539(LINC01539)	UP	USB1	U6 snRNA biogenesis phosphodiesterase 1(USB1)	UP
LRRC27	leucine rich repeat containing 27(LRRC27)	UP	UTP14C	UTP14, small subunit processome component homolog C (S. cerevisiae)(UTP14C)	UP
LRRC3B	leucine rich repeat containing 3B(LRRC3B)	UP	VARS	valyl-tRNA synthetase(VARS)	UP
LRRC8D	leucine rich repeat containing 8 family member D(LRRC8D)	UP	WRNIP1	Werner helicase interacting protein 1(WRNIP1)	UP
LY6K	lymphocyte antigen 6 complex, locus K(LY6K)	UP	YIF1A	Yip1 interacting factor homolog A, membrane trafficking protein(YIF1A)	UP
MACROD2-IT	MACROD2 intronic transcript 1(MACROD2-IT1)	UP	ZC2HC1C	zinc finger C2HC-type containing 1C(ZC2HC1C)	UP
			ZNF121	zinc finger protein 121(ZNF121)	UP
			ZNF286B	zinc finger protein 286B(ZNF286B)	UP

PROBE	NAME	CHANGE	PROBE	NAME	CHANGE	PROBE	NAME	CHANGE	PROBE	NAME	CHANGE	PROBE	NAME	CHANGE
A4GALT	alpha 1,4-galactosyltransferase(A4GALT)	DOWN	FAM102A	family with sequence similarity 102 member A(FAM102A)	DOWN	LPAR4	lysophosphatidic acid receptor 4(LPAR4)	DOWN	PKMP4	pyruvate kinase, muscle pseudogene 4(PKMP4)	DOWN	SLC13A4	solute carrier family 13 member 4(SLC13A4)	DOWN
ABCD4	ATP binding cassette subfamily D member 4(ABCD4)	DOWN	FAM136BP	family with sequence similarity 136 member B, pseudogen	DOWN	LRP4-AS1	LRP4 antisense RNA 1(LRP4-AS1)	DOWN	PLCG1	phospholipase C gamma 1(PLCG1)	DOWN	SLC25A39P1	solute carrier family 25 member 39 pseudogene 1(SLC25A3	DOWN
ABHD2	abhydrolase domain containing 2(ABHD2)	DOWN	FAM84A	family with sequence similarity 84 member A(FAM84A)	DOWN	LRRN3	leucine rich repeat neuronal 3(LRRN3)	DOWN	PLP1	proteolipid protein 1(PLP1)	DOWN	SNHG7	small nucleolar RNA host gene 7(SNHG7)	DOWN
ACTBP1	actin, beta pseudogene 1(ACTBP1)	DOWN	FAXDC2	fatty acid hydroxylase domain containing 2(FAXDC2)	DOWN	LSM3P5	LSM3 homolog, U6 small nuclear RNA and mRNA degradat	DOWN	PMEL	premelanosome protein(PMEL)	DOWN	SNORA21	small nucleolar RNA, H/ACA box 21(SNORA21)	DOWN
ACTC1	actin, alpha, cardiac muscle 1(ACTC1)	DOWN	FBXO25	F-box protein 25(FBXO25)	DOWN	MAL2	mal, T-cell differentiation protein 2 (gene/pseudogene)(M	DOWN	POT1-AS1	POT1 antisense RNA 1(POT1-AS1)	DOWN	SNORA48	small nucleolar RNA, H/ACA box 48(SNORA48)	DOWN
ACTG2	actin, gamma 2, smooth muscle, enteric(ACTG2)	DOWN	FBXO36	F-box protein 36(FBXO36)	DOWN	MALL	mal, T-cell differentiation protein like(MALL)	DOWN	POU5F1P6	POU class 5 homeobox 1 pseudogene 6(POU5F1P6)	DOWN	SNORA53	small nucleolar RNA, H/ACA box 53(SNORA53)	DOWN
ADAMTS12	ADAM metallopeptidase with thrombospondin type 1 moti	DOWN	FCF1P6	FCF1 pseudogene 6(FCF1P6)	DOWN	MED28	mediator complex subunit 28(MED28)	DOWN	PPP1R2P2	protein phosphatase 1 regulatory inhibitor subunit 2 pseud	DOWN	SNORD111	small nucleolar RNA, C/D box 111(SNORD111)	DOWN
ADCY5	adenylate cyclase 5(ADCY5)	DOWN	FGD2	FYVE, RhoGEF and PH domain containing 2(FGD2)	DOWN	METAP1D	methionyl aminopeptidase type 1D, mitochondrial(METAP	DOWN	PRELID3A	PRELI domain containing 3A(PRELID3A)	DOWN	SNORD14E	small nucleolar RNA, C/D box 14E(SNORD14E)	DOWN
AK5	adenylate kinase 5(AK5)	DOWN	FGF12	fibroblast growth factor 12(FGF12)	DOWN	METTL16	methyltransferase like 16(METTL16)	DOWN	PRNT	prion protein (testis specific)(PRNT)	DOWN	SNORD21	small nucleolar RNA, C/D box 21(SNORD21)	DOWN
AKAIN1	A-kinase anchor inhibitor 1(AKAIN1)	DOWN	FOXI1	forkhead box I1(FOXI1)	DOWN	MGAT1	mannosyl (alpha-1,3-)-glycoprotein beta-1,2-N-acetylgluco	DOWN	PRRX1	paired related homeobox 1(PRRX1)	DOWN	SNRK-AS1	SNRK antisense RNA 1(SNRK-AS1)	DOWN
AMIGO3	adhesion molecule with Ig like domain 3(AMIGO3)	DOWN	FPGT-TNNI3K	FPGT-TNNI3K readthrough(FPGT-TNNI3K)	DOWN	MIR154	microRNA 154(MIR154)	DOWN	PRSS37	protease, serine 37(PRSS37)	DOWN	SNRPEP7	small nuclear ribonucleoprotein polypeptide E pseudogene	DOWN
APEX1	apurinic/apyrimidinic endodeoxyribonuclease 1(APEX1)	DOWN	FPR1	formyl peptide receptor 1(FPR1)	DOWN	MIR202HG	MIR202 host gene(MIR202HG)	DOWN	PSMA6P4	proteasome subunit alpha 6 pseudogene 4(PSMA6P4)	DOWN	SP7	Sp7 transcription factor(SP7)	DOWN
ARHGAP19	Rho GTPase activating protein 19(ARHGAP19)	DOWN	FXYD7	FXYD domain containing ion transport regulator 7(FXYD7)	DOWN	MIR208B	microRNA 208b(MIR208B)	DOWN	PSMC1P2	proteasome 26S subunit, ATPase 1 pseudogene 2(PSMC1P	DOWN	SPINK2	serine peptidase inhibitor, Kazal type 2(SPINK2)	DOWN
ARRDC4	arrestin domain containing 4(ARRDC4)	DOWN	GABBR1	gamma-aminobutyric acid type B receptor subunit 1(GABB	DOWN	MIR2117HG	MIR2117 host gene(MIR2117HG)	DOWN	PTAFR	platelet activating factor receptor(PTAFR)	DOWN	SPRR2D	small proline rich protein 2D(SPRR2D)	DOWN
ATP6V1E2	ATPase H+ transporting V1 subunit E2(ATP6V1E2)	DOWN	GARSP1	glycyl-tRNA synthetase pseudogene 1(GARSP1)	DOWN	MIR23C	microRNA 23c(MIR23C)	DOWN	PTGES3L	prostaglandin E synthase 3 like(PTGES3L)	DOWN	SSTR2	somatostatin receptor 2(SSTR2)	DOWN
AZGP1	alpha-2-glycoprotein 1, zinc-binding(AZGP1)	DOWN	GLOD5	glyoxalase domain containing 5(GLOD5)	DOWN	MIR362	microRNA 362(MIR362)	DOWN	PTH	parathyroid hormone(PTH)	DOWN	ST8SIA1	ST8 alpha-N-acetyl-neuraminide alpha-2,8-sialyltransferase	DOWN
BEX5	brain expressed X-linked 5(BEX5)	DOWN	GP6	glycoprotein VI platelet(GP6)	DOWN	MIR3679	microRNA 3679(MIR3679)	DOWN	R3HDM2P1	R3H domain containing 2 pseudogene 1(R3HDM2P1)	DOWN	STAG3L3	stromal antigen 3-like 3 (pseudogene)(STAG3L3)	DOWN
BHLHE40-AS1	BHLHE40 antisense RNA 1(BHLHE40-AS1)	DOWN	GPER1	G protein-coupled estrogen receptor 1(GPER1)	DOWN	MIR3689D2	microRNA 3689d-2(MIR3689D2)	DOWN	RABL3	RAB, member of RAS oncogene family like 3(RABL3)	DOWN	SULT1C2	sulfotransferase family 1C member 2(SULT1C2)	DOWN
BMS1P1	Ribosome Biogenesis Factor Pseudogene	DOWN	GPRC5B	G protein-coupled receptor class C group 5 member B(GPR	DOWN	MIR3942	microRNA 3942(MIR3942)	DOWN	RAG1	recombination activating 1(RAG1)	DOWN	TANGO6	transport and golgi organization 6 homolog(TANGO6)	DOWN
C14orf105	chromosome 14 open reading frame 105(C14orf105)	DOWN	GSTM3	glutathione S-transferase mu 3(GSTM3)	DOWN	MIR4257	microRNA 4257(MIR4257)	DOWN	RALY	RALY heterogeneous nuclear ribonucleoprotein(RALY)	DOWN	TGM5	transglutaminase 5(TGM5)	DOWN
C19orf45	chromosome 19 open reading frame 45(C19orf45)	DOWN	HHLA1	HERV-H LTR-associating 1(HHLA1)	DOWN	MIR548V	microRNA 548v(MIR548V)	DOWN	RAPGEF5	Rap guanine nucleotide exchange factor 5(RAPGEF5)	DOWN	THUMPD3P1	THUMP domain containing 3 pseudogene 1(THUMPD3P1)	DOWN
C1QTNF3-AMACR	C1QTNF3-AMACR readthrough (NMD candidate)(C1QTNF3	DOWN	HIST1H2BPS2	histone cluster 1 H2B pseudogene 2(HIST1H2BPS2)	DOWN	MIR7-2	microRNA 7-2(MIR7-2)	DOWN	RASAL2-AS1	RASAL2 antisense RNA 1(RASAL2-AS1)	DOWN	TICAM2	toll like receptor adaptor molecule 2(TICAM2)	DOWN
C5orf49	chromosome 5 open reading frame 49(C5orf49)	DOWN	HMGB3P13	high mobility group box 3 pseudogene 13(HMGB3P13)	DOWN	MIR9-3HG	MIR9-3 host gene(MIR9-3HG)	DOWN	RBP1	retinol binding protein 1(RBP1)	DOWN	TLR4	toll like receptor 4(TLR4)	DOWN
C6orf1	chromosome 6 open reading frame 1(C6orf1)	DOWN	HMGB3P17	high mobility group box 3 pseudogene 17(HMGB3P17)	DOWN	MRGPRX3	MAS related GPR family member X3(MRGPRX3)	DOWN	RBP4	retinol binding protein 4(RBP4)	DOWN	TMEM141	transmembrane protein 141(TMEM141)	DOWN
CA1	carbonic anhydrase 1(CA1)	DOWN	HMGCLL1	3-hydroxymethyl-3-methylglutaryl-CoA lyase like 1(HMGCL	DOWN	MRPL35P3	mitochondrial ribosomal protein L35 pseudogene 3(MRPL3	DOWN	REG1CP	regenerating family member 1 gamma, pseudogene(REG1C	DOWN	TMEM158	transmembrane protein 158 (gene/pseudogene)(TMEM15	DOWN
CAMKV	CaM kinase like vesicle associated(CAMKV)	DOWN	HNRNPCP4	heterogeneous nuclear ribonucleoprotein C pseudogene 4	DOWN	MRPS18AP1	mitochondrial ribosomal protein S18A pseudogene 1(MRP	DOWN	RERG	RAS like estrogen regulated growth inhibitor(RERG)	DOWN	TMEM217	transmembrane protein 217(TMEM217)	DOWN
CD163L1	CD163 molecule like 1(CD163L1)	DOWN	HPX	hemopexin(HPX)	DOWN	MRPS21P2	mitochondrial ribosomal protein S21 pseudogene 2(MRPS2	DOWN	RFPL2	ret finger protein like 2(RFPL2)	DOWN	TMEM220	transmembrane protein 220(TMEM220)	DOWN
CD74	CD74 molecule(CD74)	DOWN	HTN3	histatin 3(HTN3)	DOWN	MRPS21P8	mitochondrial ribosomal protein S21 pseudogene 8(MRPS2	DOWN	RGP1	RGP1 homolog, RAB6A GEF complex partner 1(RGP1)	DOWN	TMEM67	transmembrane protein 67(TMEM67)	DOWN
CDCP1	CUB domain containing protein 1(CDCP1)	DOWN	HYAL4	hyaluronoglucosaminidase 4(HYAL4)	DOWN	MRPS31P4	mitochondrial ribosomal protein S31 pseudogene 4(MRPS3	DOWN	RHOBTB1	Rho related BTB domain containing 1(RHOBTB1)	DOWN	TMEM86A	transmembrane protein 86A(TMEM86A)	DOWN
CDON	cell adhesion associated, oncogene regulated(CDON)	DOWN	IFT172	intraflagellar transport 172(IFT172)	DOWN	MTCO1P22	mitochondrially encoded cytochrome c oxidase I pseudoge	DOWN	RN7SKP70	RNA, 7SK small nuclear pseudogene 70(RN7SKP70)	DOWN	TMPRSS4	transmembrane protease, serine 4(TMPRSS4)	DOWN
CDS2	CDP-diacylglycerol synthase 2(CDS2)	DOWN	IGF1	insulin like growth factor 1(IGF1)	DOWN	MTCO1P6	mitochondrially encoded cytochrome c oxidase I pseudoge	DOWN	RN7SL100P	RNA, 7SL, cytoplasmic 100, pseudogene(RN7SL100P)	DOWN	TPRG1	tumor protein p63 regulated 1(TPRG1)	DOWN
CEBPG	CCAAT/enhancer binding protein gamma(CEBPG)	DOWN	IL13RA2	interleukin 13 receptor subunit alpha 2(IL13RA2)	DOWN	MTCO3P7	mitochondrially encoded cytochrome c oxidase III pseudog	DOWN	RNF165	ring finger protein 165(RNF165)	DOWN	TRAPPC9	trafficking protein particle complex 9(TRAPPC9)	DOWN
CERS3	ceramide synthase 3(CERS3)	DOWN	IL17RA	interleukin 17 receptor A(IL17RA)	DOWN	MTCYBP17	mitochondrially encoded cytochrome b pseudogene 17(MT	DOWN	RNU6-656P	RNA, U6 small nuclear 656, pseudogene(RNU6-656P)	DOWN	TRIM34	tripartite motif containing 34(TRIM34)	DOWN
CHRNA3	cholinergic receptor nicotinic alpha 3 subunit(CHRNA3)	DOWN	IQSEC3	IQ motif and Sec7 domain 3(IQSEC3)	DOWN	MTND1P14	mitochondrially encoded NADH:ubiquinone oxidoreductas	DOWN	RPEP4	ribulose-5-phosphate-3-epimerase pseudogene 4(RPEP4)	DOWN	TRIM4	tripartite motif containing 4(TRIM4)	DOWN
CLEC5A	C-type lectin domain family 5 member A(CLEC5A)	DOWN	IRF1	interferon regulatory factor 1(IRF1)	DOWN	MTND1P6	mitochondrially encoded NADH:ubiquinone oxidoreductas	DOWN	RPL12P25	ribosomal protein L12 pseudogene 25(RPL12P25)	DOWN	TRIM49	tripartite motif containing 49(TRIM49)	DOWN
CORO1A	coronin 1A(CORO1A)	DOWN	JAML	junction adhesion molecule like(JAML)	DOWN	MTND2P31	mitochondrially encoded NADH:ubiquinone oxidoreductas	DOWN	RPL23AP30	ribosomal protein L23a pseudogene 30(RPL23AP30)	DOWN	TRIOBP	TRIO and F-actin binding protein(TRIOBP)	DOWN
CPEB2	cytoplasmic polyadenylation element binding protein 2(CP	DOWN	KIAA0100	KIAA0100(KIAA0100)	DOWN	MTND6P11	mitochondrially encoded NADH:ubiquinone oxidoreductas	DOWN	RPL23AP66	ribosomal protein L23a pseudogene 66(RPL23AP66)	DOWN	TTC39B	tetratricopeptide repeat domain 39B(TTC39B)	DOWN
CPM	carboxypeptidase M(CPM)	DOWN	KIAA1671	KIAA1671(KIAA1671)	DOWN	MYDGF	myeloid derived growth factor(MYDGF)	DOWN	RPL7AP57	ribosomal protein L7a pseudogene 57(RPL7AP57)	DOWN	TTLL13P	tubulin tyrosine ligase like 13, pseudogene(TTLL13P)	DOWN
CXCR2	C-X-C motif chemokine receptor 2(CXCR2)	DOWN	KIDINS220	kinase D-interacting substrate 220kDa(KIDINS220)	DOWN	MYLK-AS1	MYLK antisense RNA 1(MYLK-AS1)	DOWN	RPL9P16	ribosomal protein L9 pseudogene 16(RPL9P16)	DOWN	USP18	ubiquitin specific peptidase 18(USP18)	DOWN
CYP4B1	cytochrome P450 family 4 subfamily B member 1(CYP4B1)	DOWN	KLK4	kallikrein related peptidase 4(KLK4)	DOWN	NACAP2	nascent polypeptide associated complex alpha subunit pse	DOWN	RPS10P27	ribosomal protein S10 pseudogene 27(RPS10P27)	DOWN	USP5	ubiquitin specific peptidase 5(USP5)	DOWN
DEFB134	defensin beta 134(DEFB134)	DOWN	KRBOX1	KRAB box domain containing 1(KRBOX1)	DOWN	NDUFB4P1	NADH:ubiquinone oxidoreductase subunit B4 pseudogene	DOWN	RPS27AP6	ribosomal protein S27a pseudogene 6(RPS27AP6)	DOWN	VDAC2P2	voltage dependent anion channel 2 pseudogene 2(VDAC2P	DOWN
DPYSL5	dihydropyrimidinase like 5(DPYSL5)	DOWN	KRT4	keratin 4(KRT4)	DOWN	NOTCH2P1	notch 2 pseudogene 1(NOTCH2P1)	DOWN	RTEL1-TNFRSF	RTEL1-TNFRSF6B readthrough (NMD candidate)(RTEL1-TNF	DOWN	VHL	von Hippel-Lindau tumor suppressor(VHL)	DOWN
DUBR	DPPA2 upstream binding RNA(DUBR)	DOWN	KRTAP10-9	keratin associated protein 10-9(KRTAP10-9)	DOWN	NPM1P19	nucleophosmin 1 pseudogene 19(NPM1P19)	DOWN	S100A7A	S100 calcium binding protein A7A(S100A7A)	DOWN	VTCN1	V-set domain containing T cell activation inhibitor 1(VTCN1	DOWN
EFEMP1	EGF containing fibulin like extracellular matrix protein 1(EF	DOWN	LAMTOR3P2	late endosomal/lysosomal adaptor, MAPK and MTOR activ	DOWN	NPM1P4	nucleophosmin 1 pseudogene 4(NPM1P4)	DOWN	S100B	S100 calcium binding protein B(S100B)	DOWN	WDYHV1	WDYHV motif containing 1(WDYHV1)	DOWN
ELAVL4	ELAV like RNA binding protein 4(ELAVL4)	DOWN	LCMT1	leucine carboxyl methyltransferase 1(LCMT1)	DOWN	NPY2R	neuropeptide Y receptor Y2(NPY2R)	DOWN	SDC1	syndecan 1(SDC1)	DOWN	WFDC10B	WAP four-disulfide core domain 10B(WFDC10B)	DOWN
EML6	echinoderm microtubule associated protein like 6(EML6)	DOWN	LCN10	lipocalin 10(LCN10)	DOWN	OGFOD2	2-oxoglutarate and iron dependent oxygenase domain con	DOWN	SEC22C	SEC22 homolog C, vesicle trafficking protein(SEC22C)	DOWN	ZBED3-AS1	ZBED3 antisense RNA 1(ZBED3-AS1)	DOWN
ENOX1-AS1	ENOX1 antisense RNA 1(ENOX1-AS1)	DOWN	LCP2	lymphocyte cytosolic protein 2(LCP2)	DOWN	OR2G1P	olfactory receptor family 2 subfamily G member 1 pseudog	DOWN	SEC24C	SEC24 homolog C, COPII coat complex component(SEC24C	DOWN	ZFP62	ZFP62 zinc finger protein(ZFP62)	DOWN
ENPEP	glutamyl aminopeptidase(ENPEP)	DOWN	LECT2	leukocyte cell derived chemotaxin 2(LECT2)	DOWN	OR2H1	olfactory receptor family 2 subfamily H member 1(OR2H1)	DOWN	SEMA4B	semaphorin 4B(SEMA4B)	DOWN	ZNF287	zinc finger protein 287(ZNF287)	DOWN
EOMES	eomesodermin(EOMES)	DOWN	LGI1	leucine rich glioma inactivated 1(LGI1)	DOWN	OR4K12P	olfactory receptor family 4 subfamily K member 12 pseudo	DOWN	SEPT7P9	septin 7 pseudogene 9(SEPT7P9)	DOWN	ZNF300	zinc finger protein 300(ZNF300)	DOWN
ERVFRD-1	endogenous retrovirus group FRD member 1(ERVFRD-1)	DOWN	LINC00701	long intergenic non-protein coding RNA 701(LINC00701)	DOWN	OSGEP	O-sialoglycoprotein endopeptidase(OSGEP)	DOWN	SFTPA3P	surfactant protein A3, pseudogene(SFTPA3P)	DOWN	ZNF528	zinc finger protein 528(ZNF528)	DOWN
ETFBKMT	electron transfer flavoprotein beta subunit lysine methyltr	DOWN	LINC00939	long intergenic non-protein coding RNA 939(LINC00939)	DOWN	PAFAH2	platelet activating factor acetylhydrolase 2(PAFAH2)	DOWN	SH3PXD2B	SH3 and PX domains 2B(SH3PXD2B)	DOWN	ZNF658B	zinc finger protein 658B (pseudogene)(ZNF658B)	DOWN
ETV3	ETS variant 3(ETV3)	DOWN	LINC01128	long intergenic non-protein coding RNA 1128(LINC01128)	DOWN	PATJ	PATJ, crumbs cell polarity complex component(PATJ)	DOWN	SHISA9	shisa family member 9(SHISA9)	DOWN	ZNF663P	zinc finger protein 663, pseudogene(ZNF663P)	DOWN
EYA1	EYA transcriptional coactivator and phosphatase 1(EYA1)	DOWN	LMBR1L	limb development membrane protein 1 like(LMBR1L)	DOWN	PHF7	PHD finger protein 7(PHF7)	DOWN	SIRT7	sirtuin 7(SIRT7)	DOWN	ZNF726	zinc finger protein 726(ZNF726)	DOWN
												ZNF773	zinc finger protein 773(ZNF773)	DOWN
												ZNF781	zinc finger protein 781(ZNF781)	DOWN
												ZNF821	zinc finger protein 821(ZNF821)	DOWN
												9-Mar	membrane-associated ring finger (C3HC4) 9	DOWN

Supplementary Table 2. RAB25_UP and RAB25_DOWN gene sets. Gene symbols and descriptions for genes identified as significantly increased (*top, red*) and decreased (*bottom, blue*) in RAB25-expressing vs. pcDNA3 control HEY ovarian cancer cells.

Peptide	Sequence	Retention Time (min)	m/z expected	/ (m/z found)
fRFP1	FITC-(PEG)-KKEFQVRELEDYIDNLLVRVMEETPNILRIPA	8.0	1469.4, 1102.3	/(1469.3, 1102.2)
fRFP2	FITC-(PEG)-ERDTHIRELEDYIDNLLVRVMEETPSILRVPR	8.0	1483.7, 1113.0	/(1483.6, 1112.9)
fRFP3	FITC-(PEG)-KQEEINFRLQDYIDRIIVAIMETNPSILEVK	7.8	1418.3, 1064.0	/(1418.2, 1064.1)
fRFP4	FITC-(PEG)-EQEEINFRLRQYMDKIILAILDHNPSILEIKH	7.5	1485.4, 1114.3	/(1485.3, 1114.3)
fRFP5	FITC-(PEG)-KQEEINFRLQDYIDRIIVAIM_{ox}ETNPSILEVK	7.1	1423.6, 1068.0	/(1423.6, 1068.0)
fRFP6	FITC-(PEG)-βRQVRELES_5YIDS_5LLVRVMEETPNILRIPR	9.1	1423.6, 1068.0	/(1423.6, 1068.0)
fRFP7	FITC-(PEG)-βRQVRELEDYIDS_5LLVS_5VMEETPNILRIPR	9.1	1389.3, 1042.2	/(1389.2, 1042.2)
fRFP8	FITC-(PEG)-βRQVRELEDYIDRLLVS_5VMES_5TPNILRIPR	9.6	1398.3, 1049.0	/(1398.2, 1049.0)
fRFP9	FITC-(PEG)-βRQVRELEDS_5IDRS_5LVRVMEETPNILRIPR	8.6	1401.3, 1051.2	/(1401.3, 1051.2)
fRFP10	FITC-(PEG)-βRINFRLQS_5YIDS_5IIVAIMETNPSILEVK	8.3	1343.6, 1007.9	/(1343.3, 1007.9)
fRFP11	FITC-(PEG)-βRINFRLQDYIDS_5IIVS_5IMETNPSILEVK	8.6	1330.5, 998.2	/(1330.0, 998.2)
fRFP12	FITC-(PEG)-βRINFRLQDYIDRIIVS_5IMES_5NPSILEVK	8.4	1348.9, 1011.9	/(1348.3, 1012.2)
fRFP13	FITC-(PEG)-βRINFRLQDS_5IDRS_5IVAIMETNPSILEVK	8.0	1351.6, 1014.0	/(1351.3, 1013.9)
bRFP14	biot-PEG-WβRQVRELENYIDRLLVS_5VN_LES_5TPNILRIPR	9.7	1400.0, 1050.3	/(1399.5, 1050.1)
bRFP24	biot-PEG-WβRINFRLQNS_5IDRS_5IVRIFETNPSILRVK	7.7	1364.3, 1023.5	/(1363.9, 1023.2)
bRFP26	biot-PEG-WβRINFRLQNS_5IDRS_5IVRIN_LETNPSILRVK	7.7	1353.0, 1015.0	/(1352.6, 1014.6)
bRFP31	biot-PEG-WβRINFRLQNS_5IDRS_5AARIAETNPSILEVK	7.2	1306.5, 980.2	/(1306.0, 979.8)
fRFP14	FITC-PEG-βRQVRELENYIDRLLVS_5VN_LES_5TPNILRIPR	15.9*	1392.0, 1044.2	/(1391.9, 1044.1)
fRFP24	FITC-PEG-βRINFRLQNS_5IDRS_5IVRIFETNPSILRVK	15.8*	1356.6, 1017.7	/(1356.3, 1017.4)
fRFP26	FITC-PEG-βRINFRLQNS_5IDRS_5IVRIN_LETNPSILRVK	15.6*	1344.6, 1008.7	/(1344.9, 1008.8)
fRFP31	FITC-PEG-βRINFRLQNS_5IDRS_5AARIAETNPSILEVK	15.0*	1298.2, 973.9	/(1298.5, 974.3)
fRFP32	FITC-PEG-βRINFRLQNS_5IDRS_5AARIAETNPSILRVK	14.8*	1308.9, 981.9	/(1307.5, 980.7)
RFP4	W-βEQEEINFRLRQYMDKIILAILDHNPSILEIKH	13.9*	1393.3, 1045.2	/(1392.4, 1044.7)
RFP14	W-βRQVRELENYIDRLLVS_5VN_LES_5TPNILRIPR	14.5*	1276.2, 957.4	/(1276.1, 957.3)
RFP24	W-βRINFRLQNS_5IDRS_5IVRIFETNPSILRVK	14.3*	1240.5, 930.6	/(1240.1, 930.4)
RFP26	W-βRINFRLQNS_5IDRS_5IVRIN_LETNPSILRVK	14.0*	1229.2, 922.1	/ (1229.0, 922.0)
RFP31	W-βRINFRLQNS_5IDRS_5AARIAETNPSILEVK	13.4*	1182.7, 887.3	/ (1182.4, 887.0)
RFP32	W-βRINFRLQNS_5IDRS_5AARIAETNPSILRVK	13.2*	1192.3, 894.5	/ (1191.8, 894.0)

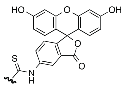

FITC-

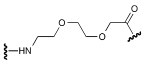

-PEG-

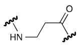

-beta-alanine-

Supplementary Table 3. Name, sequence and characteristic LC-MS data for peptides used in this study. Residues highlighted in yellow denote potential gain-of-function mutations, those in green denote loss-of-function alanine replacement for negative controls, and red denotes S_5 amino acid residues incorporated for peptide stapling. Chemical structures for linkers and fluorescent tags are shown. The first series of RFP peptides (RFP1-RFP13) were analyzed by LC/MS using a C18 reverse-phase column (Agilent, 2.1 x 150 mm, pore size 80 A, particle size 3.5 μm); Buffer A (H_2O/0.1% TFA) and Buffer B (ACN/0.1% TFA); and a 15 minute method with the following gradient (flow rate 0.5 mL/min): 10-100% buffer B over 10 min, 100% buffer B for 2 min, 100-10% buffer B over 1 min, and 10% buffer B over 2 min. Optimized RFP peptides (denoted by * on retention time) were analyzed by LC/MS using a C18 reverse-phase

column (Phenomenex, 5.0 x 50 mm, pore size 110 A, particle size 5µm); Buffer A (5/95/0.1% ACN/H2O/TFA) and Buffer B (95:5:0.1% ACN/H2O/TFA); and a 20 minute method with the following gradient (flow rate 0.5 mL/min): 0% buffer B over 3 min, 0-65% buffer B over 15 min, 65-100% buffer B over 1 min; 100-0% buffer B over 1 min.

Gene name	ID	Forward Primer	Reverse Primer
AMIGO3	**NM_198722.2**	caagaacggcctctacttgc	aggccaagcatacgtactcg
CEBPG	**NM_001252296.1**	catggatcgaaacagtgacg	gcttccaaccgttcattctc
CXCR2	**NM_001168298.1**	cgctccgtcactgatgtcta	aaatccagccattcaccttg
FOXI1	**NM_012188.4**	cctctagcacagcctccttg	gaggctccatccaagatgtc
FPR1	**NM_001193306.1**	tattgccaccaagatccaca	gatatggggaccagcagaga
GPER1	**NM_001039966.1**	agggacaagctgaggctgta	gctgaacctcacatccgact
IGF1	**NM_000618.4**	tctcttctacctggcgctgt	cacgaactgaagagcatcca
LPAR4	**NM_001278000.1**	accttgtgccttgcaactct	ggctttgtggtcaaaggtgt
LY6K	**NM_001160354.1**	aaggaggtgcaaatggacag	cctgtgtgttgaatgctgct
MGAT1	**NM_001114617.1**	ccagctggacctgtcttacc	cggtcattggtcctcacttt
P2RY14	**NM_001081455.1**	cctccagatgaatcctgctc	agagctgggcacgtaaaaga
PRSS23	**NM_001293178.1**	caggtgggcatctacatcct	gtccttcccaaaaatgctga
RAB25	**NM_020387.3**	ccctcctggtgtttgaccta	tttgttacccacgagcatga
DNAJA3	**NM_001135110.2**	aggacaagccaagcagaaaa	tgcaccctgaacgtaatgaa
VHL	**NM_000551.3**	aggtcacctttggctcttca	ttggcaaaaataggctgtcc
ATCB	**NM_001101.3**	ctcttccagccttccttcct	agcactgtgttggcgtacag
18S		TaqMan	Assay ID: Hs99999901_s1

Supplementary Table 4. Primer sequences used for qPCR analysis.
